# Metabolic Syndrome Induces Epigenetic Alterations in Mitochondria-Related Genes in Swine Mesenchymal Stem Cells

**DOI:** 10.3390/cells12091274

**Published:** 2023-04-27

**Authors:** Kamalnath S. Rajagopalan, Sara Kazeminia, Logan M. Glasstetter, Rahele A. Farahani, Xiang-Yang Zhu, Hui Tang, Kyra L. Jordan, Alejandro R. Chade, Amir Lerman, Lilach O. Lerman, Alfonso Eirin

**Affiliations:** 1 Division of Nephrology and Hypertension, Mayo Clinic, Rochester, MN 55905, USA; 2 Division of Endocrinology, Diabetes, Metabolism and Nutrition, Mayo Clinic, Rochester, MN 55905, USA; 3 Department of Medical Pharmacology and Physiology and Department of Medicine, University of Missouri-Columbia, Columbia, MO 65211, USA; 4 Department of Cardiovascular Diseases, Mayo Clinic, Rochester, MN 55905, USA

**Keywords:** mesenchymal stem cells, metabolic syndrome, mitochondria, epigenetics, MeDIP-seq, DNA methylation

## Abstract

Autologous mesenchymal stem/stromal cells (MSCs) have demonstrated important therapeutic effects in several diseases. Cardiovascular risk factors may impair MSC mitochondrial structure and function, but the underlying mechanisms remain unknown. We hypothesized that metabolic syndrome (MetS) induces epigenetic alterations in mitochondria-related genes in swine MSCs. Pigs were fed a Lean or MetS diet (*n* = 6 each) for 16 weeks. MSCs were collected from subcutaneous abdominal fat, and DNA hydroxymethylation (5 hmC) profiles of mitochondria-related genes (MitoCarta-2.0) were analyzed by hydroxymethylated DNA immunoprecipitation and next-generation sequencing (hMeDIP-seq) in Lean- and MetS-MSCs untreated or treated with the epigenetic modulator vitamin (Vit)-C (*n* = 3 each). Functional analysis of genes with differential 5 hmC regions was performed using DAVID6.8. Mitochondrial structure (electron microscopy), oxidative stress, and membrane potential were assessed. hMeDIP-seq identified 172 peaks (associated with 103 mitochondrial genes) with higher and 416 peaks (associated with 165 mitochondrial genes) with lower 5 hmC levels in MetS-MSCs versus Lean-MSCs (≥2-fold, *p* < 0.05). Genes with higher 5 hmC levels in MetS + MSCs were primarily implicated in fatty acid metabolism, whereas those with lower 5 hmC levels were associated with electron transport chain activity. Vit-C increased 5 hmC levels in mitochondrial antioxidant genes, improved mitochondrial structure and membrane potential, and decreased oxidative stress. MetS alters 5 hmC levels of mitochondria-related genes in swine MSCs. Vit-C modulated 5 hmC levels in these genes and preserved mitochondrial structure and function in MetS-MSCs. These observations may contribute to development of strategies to overcome the deleterious effects of MetS on MSCs.

## 1. Introduction

Transplantation of autologous mesenchymal stem/stromal cells (MSCs) has exhibited successful therapeutic benefits for several diseases. These multipotent stem cells possess capacities for self-renewal and multidirectional differentiation [1], being able to be isolated in large amounts from several tissues, including adipose tissue [2]. Importantly, MSCs have been proven to be safe and effective for ameliorating tissue injury and promoting functional recovery in experimental studies [3,4,5,6] and clinical trials [7,8]. 

Despite the regenerative potential of adipose-tissue-derived MSCs, their therapeutic efficacy is limited by cardiovascular risk factors, which may compromise the functionality of these cells [9,10,11]. Experimental metabolic syndrome (MetS), which encompasses several cardiovascular risk factors, such as obesity, hypertension, insulin resistance, and hyperlipidemia, is associated with increased inflammation and senescence [12], and it impairs the viability and differentiation of adipose tissue-derived MSCs [13,14]. 

Interestingly, MetS-induced MSC dysfunction is associated with structural and functional damage in mitochondria [15,16], which supply energy and modulate important cellular functions, including production of reactive oxygen species (ROS), proliferation, and apoptosis [17]. We have previously shown that diet-induced MetS in swine instigates MSC mitochondrial swelling and cristae remodeling, as well as decreased production of ATP, which is associated with changes in the expression of mitochondria-related genes [15,18]. However, the mechanisms by which MetS modulates the mitochondrial transcriptome and their impact on MSC mitochondrial morphology and function remain largely unknown. 

Epigenetic changes, which refer to alterations in the cellular gene expression profile without changes in the DNA sequence [19], are important for maintaining the immunomodulatory function of MSCs [20] and have been proposed to play major roles in the pathogenesis of MetS [21,22]. 5-Hydroxymethylcytosine (5 hmC) is a stable epigenetic mark generated during oxidation of 5-methylcytosine (5 mC) by the Ten-Eleven Translocation (TET) methylcytosine dioxygenases, accounting for up to 10% of 5 mC in stem cells [20] and regulating mitochondrial gene expression following ischemic injury [23]. However, whether MetS alters 5 hmC levels in genes encoding for mitochondrial proteins in MSCs, compromising the structure and function of these organelles, has not been explored. 

In the current study, we took advantage of a well-established diet-induced model of MetS in swine and applied hydroxy methylated DNA immunoprecipitation and next-generation sequencing (hMeDIP-seq) to test the hypothesis that MetS induces epigenetic alterations in mitochondria-related genes in swine MSCs. We further explored whether co-incubation of MetS-MSCs with the epigenetic modulator vitamin (Vit)-C [24,25] attenuates mitochondrial structural abnormalities and dysfunction. 

## 2. Materials and Methods

### 2.1. Experimental Design

Animal studies were approved by the Institutional Animal Care and Use Committee. Twelve 3-month-old female domestic pigs (Manthei Hog Farm, Elk River, MN, USA) were studied for 16 weeks. Previous studies have shown that females are more protected from the effects of MetS compared to males [26,27]. Therefore, we opted for using female pigs to test whether the deleterious effects of MetS on MSCs outweigh this gender-specific protection. At baseline, pigs were randomized into 2 groups (*n* = 6 each) and fed either a Lean diet (standard pig chow) or a MetS diet (5B4L; Purina, % kcal: 17% protein, 20% fructose, 20% complex carbohydrates, and 43% fat, supplemented with 2% cholesterol and 0.7% sodium cholate by weight) [28] for the duration of the study, with free access to water. Body weight, intra-arterial blood pressure, total cholesterol, low-density lipoprotein (LDL), triglycerides, and fasting glucose and insulin levels were obtained at the end of the study. Insulin resistance was assessed by homeostasis model assessment of insulin resistance (HOMA-IR) score: fasting insulin (microU/L) × fasting glucose (nmol/L)/22.5) [28]. Pigs were then euthanized with sodium pentobarbital (100 mg/kg IV, Fatal Plus^®^, Vortech Pharmaceuticals, Dearborn, MI, USA), and subcutaneous abdominal adipose tissue (5–10 g) was collected for MSC isolation.

### 2.2. MSC Isolation and Characterization

MSCs were isolated from swine subcutaneous abdominal fat tissue, as previously shown [5,29]. Briefly, fat tissue was digested in collagenase H, filtered through a 100 μm cell strainer, and centrifuged. Cells were then cultured for 3 weeks in advanced MEM medium (Gibco/Invitrogen, Carlsbad, CA, USA) supplemented with 5% platelet lysate (PLTmax, Mill Creek Life Sciences, Rochester, MN, USA). The third passage (p) was collected, and cellular phenotype was confirmed by expression of the MSCs markers CD44, CD73, CD90, and CD105; lack of expression of the progenitor cell marker CD34; and the common leukocyte marker CD45, as well as by their capacity for tri-lineage differentiation, as previously shown [3,5,30]. Then, Lean- and MetS-MSCs were cultured for another passage with or without in vitro co-incubation for 48 h (starting at 80–90% confluence) with 50 μg/mL of Vit-C (*n* = 6 each) [31], an epigenetic modifier that enhances TET catalytic activity [24], or dimethyl alpha-ketoglutarate (DMαKG), a co-factor that increases 5 hmC abundance [32].

### 2.3. hMeDIP-seq

hMeDIP-seq was performed as previously described [33,34] in randomly selected Lean- and MetS-MSCs untreated or treated with Vit-C (*n* = 3, each). Genomic DNA from MSC samples was extracted using the DNeasy Blood and Tissue Kit (Qiagen, Cat.#:69504) with RNase treatment, diluted to 100 ng/μL in TE buffer (NanoDrop spectrophotometer), and sonicated to produce DNA fragments with an average size of 200 bp (Pico Bioruptor, Diagenode, Seraing, Belgium). Fragmented DNA was denatured at 95 °C for 10 min and immunoprecipitated for 3 h at 4 °C with 0.5–2 µg of modification-specific antibodies against 5 hmC (EDL HMC-1A) in a final volume of 200 μL IP buffer (10 mM sodium phosphate, pH 7.0; 140 mM NaCl; 0.05% Triton X-100). Magnetic protein G Dynabeads (30 μL; Invitrogen, Cat.#100-03D) were added, and the reactions were further incubated overnight. Beads were washed three times with 1 mL of IP buffer and twice with 1 mL of 1× TE buffer. The enriched DNA fragments were eluted from the beads, purified using the ssDNA/RNA Clean and Concentrator (Zymo Research, Cat.#:D7010), and quantified with the Qubit ssDNA High Sensitivity Assay Kit (Thermo-Fisher Scientific, Waltham, MA, USA, Cat.#:Q10212). Libraries were prepared by the ACCEL-NGS^®^ 1S Plus DNA library kit (Cat.#:10024; Swift Bioscience, Ann Arbor, MI, USA) and sequenced to 51 base pairs from both ends using the Illumina HiSeq 4000 instrument (Illumina, San Diego, CA 92122 USA) at the Mayo Clinic Medical Genome Facility Sequencing Core.

Bioinformatic analysis of hMeDIP-seq data was performed as previously described [33,34]. Paired-end sequenced FASTQ files were aligned to the porcine reference genome (susScr 11.1) using bowtie2 (v2.3.3.1) [35]. Duplicate reads were removed with MarkDuplicates (PICARD v1.67), and hMeDIP-seq peaks were called using MACS2 [36]. Differential 5 hmC peak analysis was performed with the R package DiffBind (v2.14.0), using the HOMER [37] (v4.10) peak annotation tool to assign differential peaks and genomic coverage bins to the corresponding genes. The 5 hmC coverage analysis used per-base coverage of regions of interest, calculated with bedtools (v2.20.0) genomeCoverageBed. 

Genes associated with 5 hmC peaks were filtered by an online inventory of mammalian mitochondrial genes, MitoCarta 3.0 [38]. Differential 5 hmC peaks in mitochondria-related genes were determined on the basis of fold-change (MetS/Lean) ≥ 2 (high 5 hmC) or fold-change (Lean/MetS) ≥ 2 (low 5 hmC) and *p* < 0.05. Genes associated with differentially hydroxymethylated regions were sorted on the basis of whether these peaks were exclusively high or low in 5 hmC, using Venn diagram analysis (VENNY 2.1; http://bioinfogp.cnb.csic.es/tools/venny/ (accessed on 17 February 2023)). Furthermore, these genes were classified by their molecular function and protein class using Protein Analysis Through Evolutionary Relationships (PANTHER) [39]. Functional annotation clustering analysis and interrogation of protein functional and physical interactions were performed using the Database for Annotation, Visualization and Integrated Discovery (DAVID) v6.8 and the Search Tool for the Retrieval of Interacting Genes (STRING) v9.1 (http://string-db.org/ (accessed on 1 March 2023)), respectively. Representative hMeDIP-seq reads were visualized, and individual genes examined, using Integrative Genomics Viewer (IGV, Broad Institute, Cambridge, MA, USA, 02141) [40]. Analysis of the effect of Vit-C treatment on 5 hmC levels of mitochondria-related genes in Lean- and MetS-MSCs was performed in the same manner as the analysis described above for the MetS vs. lean condition. 

The overall (entire genome) 5 hmC landscape was compared between Lean-MSCs and MetS-MSCs, and between MetS-MSC and MetS-MSCs + Vit-C using hMeDIP-seq, whereas global 5 mC levels were assessed by immunofluorescence (D3S2Z, Rabbit mAb #28692–Cell Signaling, Danvers, MA, USA) staining in Lean- and MetS-MSCs untreated or treated with Vit-C. 

### 2.4. Validation of Selected Gene Expression 

Quantitative polymerase chain reaction (qPCR) using the ΔΔCt method was performed to validate expression levels of randomly selected mitochondria-related genes with differential 5 hmC peaks in p3 MetS-MSCs versus Lean-MSCs, MetS-MSCs + Vit-C versus MetS-MSCs, and MetS-MSCs+ DMαKG versus MetS-MSCs, and repeated in p-0 Lean- and MetS-MSCs. In addition, expression of the epigenetic enzymes TET1, TET2, and TET3 was assessed in Lean- and MetS-MSCs. Briefly, total RNA was extracted from cells using the kit (#AM1556, Life Technologies, Carlsbad, CA, USA). Then, SuperScript VILO cDNA synthesis kit (#11754-050) was used to obtain cDNA. Relative quantitative PCR utilized Taqman assays. All primers were from ThermoFisher Scientific (Waltham, MA, USA) (HADHA: ss03391088, ALDH5A1: ss04327492, NDUFB2: ss04322158, COX10: ss04328110, ssCYP11A1: 03384849, MCEE: ss03818732, HADHB: ss03391097, FASN: ss03386194, ETFB: ss03373707, CYB5A: ss03391607, TET1: ss03389746, TET2: ss03375629, and TET3: ss03376563). Fold change of gene expression was calculated using the 2-ΔΔCT method and gene expression was normalized to GAPDH.

### 2.5. MSC Mitochondrial Structure and Function

Mitochondrial morphology was assessed using transmission electron microscopy in Lean-MSCs and MetS-MSCs untreated or treated with Vit-C, as previously shown [41,42]. Cells were preserved in Trump’s fixative solution (4% formaldehyde and 0.1% glutaraldehyde in 0.1 M phosphate buffer) overnight at room temperature and processed at the Mayo Clinic Electron Microscopy Core. MSCs were mounted on mesh grids, stained with aqueous uranyl acetate and lead citrate, and scanned using a Phillips CM10 Transmission Electron Microscope. For analysis, we randomly selected 10 representative MSCs per sample. Mitochondrial density was assessed by counting the number of mitochondria per cell, whereas mitochondrial area (nm^2^) and matrix density (1/mean gray values) were measured in 10 representative mitochondria in these cells using ImageJ (Version 1.5, National institute of Health) [43]. Results were averaged per pig. 

Mitochondrial production of ROS was measured by Mito-SOX (2 μM for 30 min at 37 °C, ThermoFisher, Cat.#M36008) [44] and membrane potential by tetramethylrhodamine ethyl ester (TMRE, 50 nM for 20 min at 37 °C, ThermoFisher, Waltham, MA, USA, Cat.#T669) [45] in Lean-MSCs and MetS-MSCs untreated or treated with Vit-C or DMαKG. Triplicate experiments were carried out for each set. In addition, 5′ AMP-activated protein kinase (AMPK) immunoreactivity was assessed by immunofluorescence staining (Cell signaling, Cat#2532). 

### 2.6. Statistical Analysis

Statistical analysis was performed using the JMP Pro 14.0 software (SAS Institute Inc., Cary, NC, USA). Results are expressed as mean ± SD. Data distribution was assessed using the Shapiro–Wilk test. Comparisons between groups were performed using Student’s *t*-test or the Kruskal–Wallis test, as appropriate. Statistical significance was accepted for *p* < 0.05. 

## 3. Results

### 3.1. Systemic Characteristics

After 16 weeks of diet, MetS as compared with Lean pigs presented with increased body weight, blood pressure, total cholesterol, LDL-cholesterol, and triglyceride levels (Table 1). Fasting glucose levels were comparable between the groups, but fasting insulin levels and HOMA-IR score were higher in MetS versus Lean pigs, indicating successful development of pre-diabetic MetS. 

### 3.2. MetS Induced Epigenetic Changes in Mitochondria-Related Genes in MSCs 

**Figure 1:**
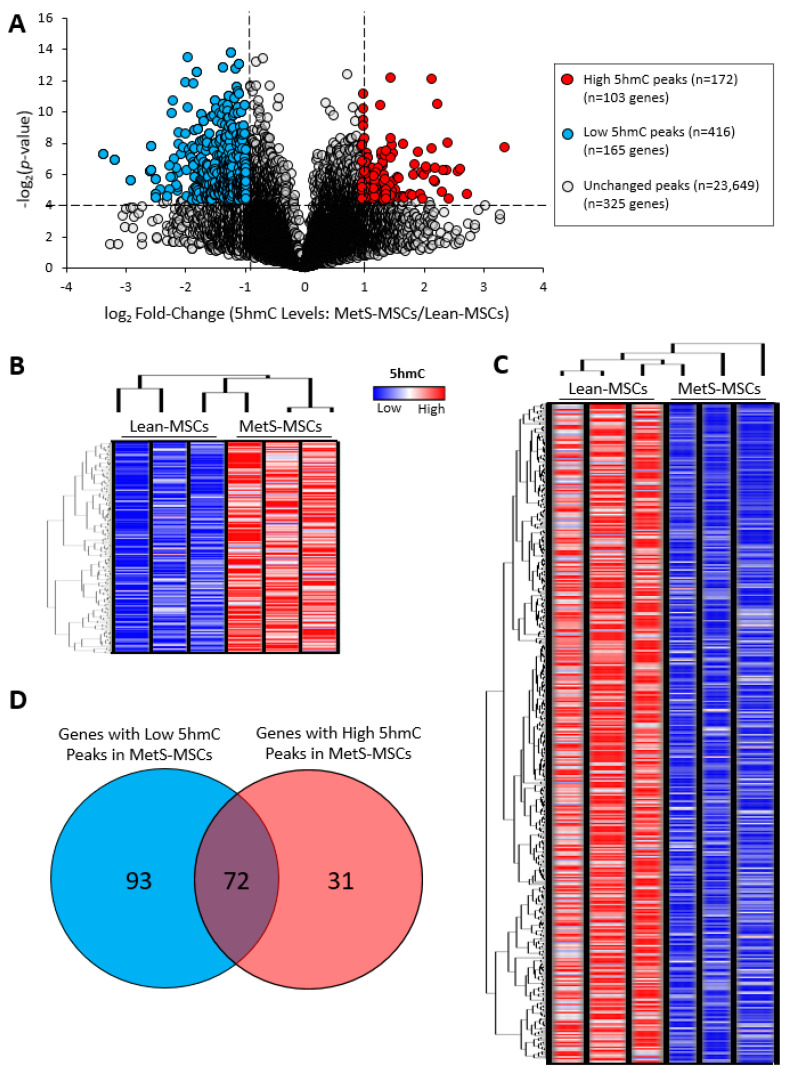
MetS induced epigenetic changes in swine adipose-tissue-derived MSCs. (**A**) Volcano plot showing 24,237 5-hydroxymethylcytosine (5 hmC) peaks in mitochondria-related genes, of which 172 were higher and 416 lower in MetS-MSCs compared to Lean-MSCs. The vertical axis (y-axis) corresponds to −log 2 (*p*-value), and the horizontal axis (x-axis) displays log 2-fold change (MetS-/Lean-MSCs). Higher (*n* = 172 peaks in 103 genes) and lower (*n* = 416 peaks in 165 genes) 5 hmC peaks in MetS- versus Lean-MSCs are shown as red and blue dots, respectively, while non-significant peaks are shown as gray dots (*p*-value <0.05 and fold changes ≥2 are indicated by black dashed lines). (**B**) Heat map representing 172 peaks in mitochondria-related genes with higher 5 hmC levels in MetS- versus Lean-MSCs (*n* = 3 each). (**C**) Heat map representing 416 peaks in mitochondria-related genes with lower 5 hmC levels in MetS- vs. Lean-MSCs (*n* = 3 each). (**D**) Venn diagram showing 31 mitochondria-related genes with exclusively higher, 93 with exclusively lower, and 72 with both higher and lower 5 hmC peaks in MetS-MSCs versus Lean-MSCs.

Analysis of high-throughput hMeDIP-seq data revealed a total of 24,237 5 hmC peaks in mitochondria-related genes of Lean- and MetS-MSCs (Figure 1A). Bioinformatic analysis identified 588 differentially-hydroxymethylated regions within these genes, including 172 hyper-hydroxymethylated peaks (Figure 1B) corresponding to 103 genes and 416 hypo-hydroxymethylated peaks (Figure 1C) corresponding to 165 genes in MetS- versus Lean-MSCs. Venn diagram analysis showed 72 mitochondrial genes featuring both hyper- and hypo-hydroxymethylated peaks in MetS- versus Lean-MSCs (Figure 1D). Consequently, we excluded those genes to focus on analyzing 31 mitochondrial genes with exclusively high 5 hmC peaks and 93 genes with exclusively low 5 hmC peaks in MetS- versus Lean-MSCs (Figure 1D).

### 3.3. Mitochondrial Genes with Exclusively High 5 hmC Peaks in MetS-MSCs 

**Figure 2:**
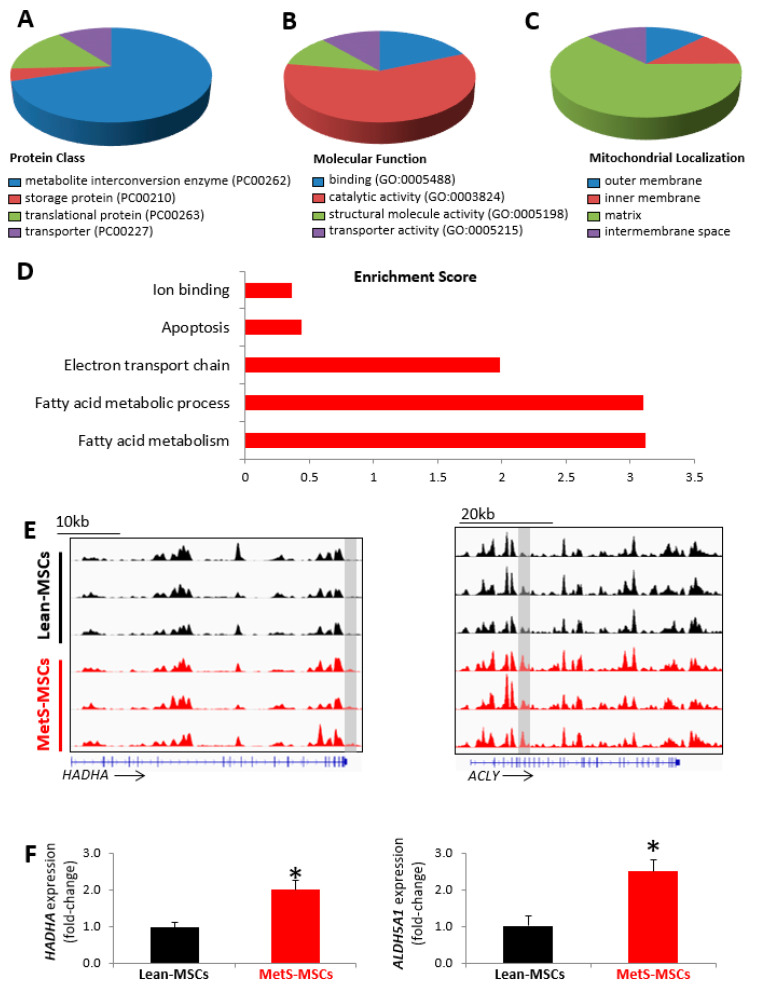
Mitochondria-related genes with high 5 hmC peaks in MetS-MSCs. Gene ontology analysis showing protein class (**A**), molecular function (**B**), mitochondrial localization (**C**), and functional annotation based on enrichment score (**D**). (**E**) Representative 5 hmC profiles for the candidate mitochondrial genes implicated in fatty acid metabolism hydroxyacyl-CoA dehydrogenase trifunctional multienzyme complex subunit alpha (*HADHA*) and ATP citrate lyase (*ACLY*) in MetS-MSCs and Lean-MSCs (Integrative Genomics Viewer). Gray rectangles indicate regions (peaks) with high 5 hmC. (**F**) Expression (qPCR) of the candidate mitochondrial genes *HADHA* and aldehyde dehydrogenase 5 family member A1 (*ALDH5A1*) was higher in MetS-MSCs versus Lean-MSCs (* *p* < 0.05 vs. Lean-MSCs) (*n* = 6 each).

Mitochondria-related genes with peaks exclusively hyper-hydroxymethylated in MetS-MSCs coded for metabolic interconversion enzymes with binding and catalytic activity, primarily localized to the mitochondrial matrix (Figure 2A–C). Functional analysis indicated that these proteins are principally implicated in fatty acid metabolism and fatty acid metabolic process, followed by electron transport chain activity, apoptosis, and ion binding (Figure 2D). Genes involved in fatty acid metabolism included hydroxyacyl-CoA dehydrogenase (*HADH*), HADH trifunctional multienzyme complex subunit alpha and beta (*HADHA* and *HADHB*, respectively), fatty acid synthase (*FASN*), ATP citrate lyase (*ACLY*), and aldehyde dehydrogenase 5 family member A1 (*ALDH5A1*), among others (Figure 2E–F), and showed many interactions (Figure S1A). Expression of the candidate genes *HADHA* and *ALDH5A1* followed the same patterns as the hMeDIP-seq findings, with higher levels both in p-3 (Figure 2F) and in p-0 (Figure S1B) MetS- versus Lean-MSCs.

### 3.4. Mitochondrial Genes with Exclusively Low 5 hmC Peaks in MetS-MSCs

**Figure 3:**
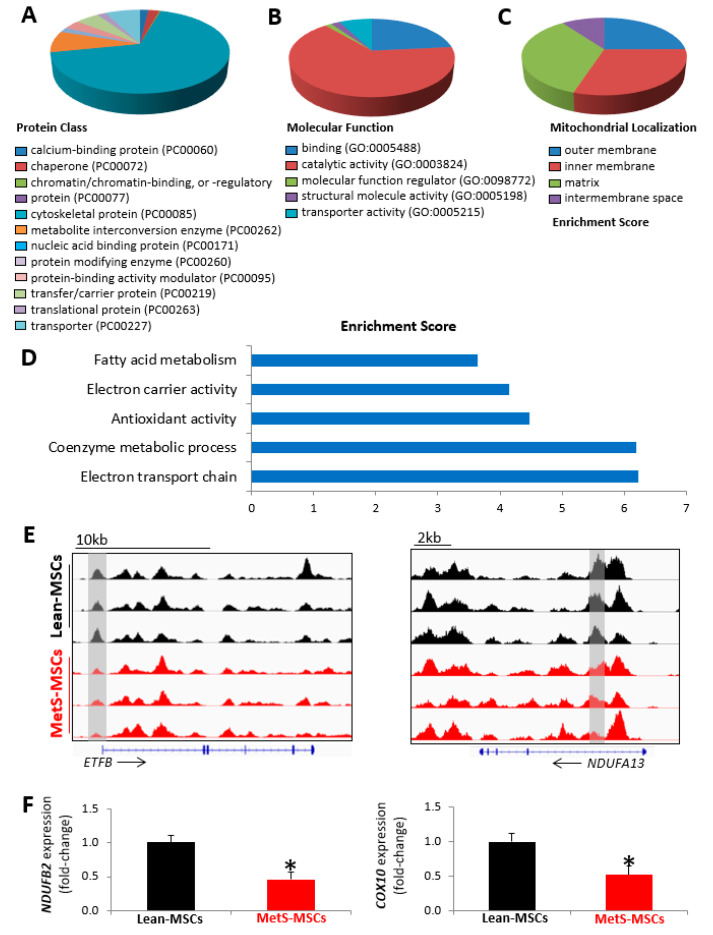
Mitochondria-related genes with low 5 hmC peaks in MetS-MSCs. Gene ontology analysis showing protein class (**A**), molecular function (**B**), mitochondrial localization (**C**), and functional annotation based on enrichment score (**D**). (**E**) Representative 5 hmC profiles for the candidate mitochondrial genes implicated in electron transport chain electron transfer flavoprotein subunit beta (*ETFB*) and NADH:ubiquinone oxidoreductase subunit A13 (*NDUFA13*) in MetS-MSCs and Lean-MSCs (Integrative Genomics Viewer). Gray rectangles indicate regions (peaks) with low 5 hmC. (**F**) Expression (qPCR) of mitochondrial genes NADH: ubiquinone oxidoreductase subunit B6 (*NDUFB2*) and cytochrome C oxidase assembly factor heme A farnesyltransferase *COX10* (*COX10*), which was lower in MetS-MSCs versus Lean-MSCs (* *p* < 0.05 vs. Lean-MSCs) (*n* = 6 each).

Mitochondrial genes with peaks exclusively hypo-hydroxymethylated in MetS-MSCs coded for cytoskeletal proteins enzymes with binding and catalytic activity, primarily distributed within the mitochondrial matrix and inner mitochondrial membrane (Figure 3A–C). Functional analysis indicated that these proteins are mainly implicated in electron transport chain activity and the coenzyme metabolic process, followed by antioxidant activity (Figure 3D). Genes involved in electron transport chain activity include NADH: ubiquinone oxidoreductase subunits A13, B2, and B6 (*NDUFA13*, *NDUFB2*, and *NDUFB6*, respectively); electron transfer flavoprotein subunit beta (ETFB); cytochrome C oxidase assembly factor heme A:farnesyltransferase COX10 (*COX10*); and ubiquinol-cytochrome C reductase, complex III subunit X (UQCR10), among others (Figure 3E,F), and showed many interactions (Figure S2A). Expression of the candidate genes *NDUFB2* and *COX10* followed the same patterns as the hMeDIP-seq findings, with lower levels both in p-3 (Figure 3F) and in p-0 (Figure S2B) in MetS- versus Lean-MSCs.

Analysis of the entire nuclear DNA genome identified a total of found 7022 hyper- and 9733 hypo-hydroxymethylated peaks (corresponding to 2142 and 2604 genes, respectively) in MetS- compared to Lean-MSCs (Figure S3A), which were primarily implicated in important cellular functions, including regulation of response to stimulus, multicellular processes, negative regulation of cell signaling, and cell death, among others (Figure S3B,C).

### 3.5. Vit-C Modulated 5 hmC Levels in Mitochondria-Related Genes in MetS-MSCs

**Figure 4:**
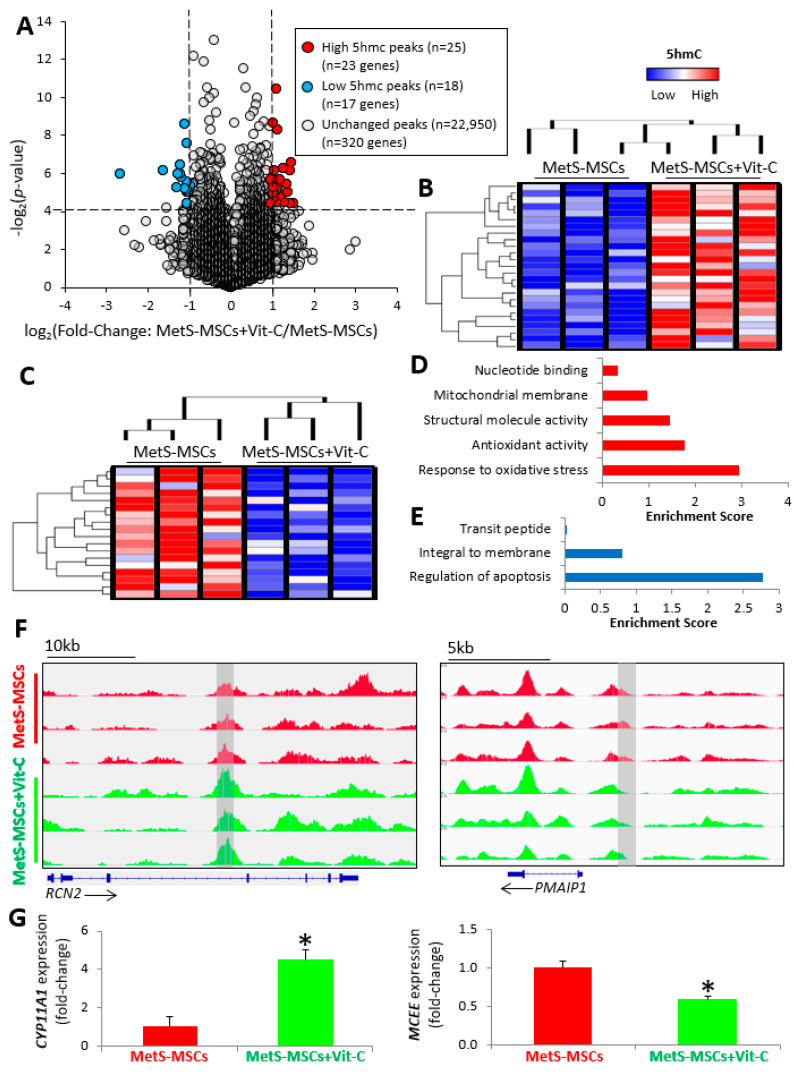
Vitamin (Vit)-C modulated 5 hmC levels in mitochondria-related genes in MetS-MSCs. (**A**) Volcano plot showing 22,993 5 hmC peaks in mitochondria-related genes identified in MetS-MSCs treated with Vit-C (50 μg/mL for 48 h) versus untreated MSCs (MetS-MSCs + Vit-C/MetS-MSCs). The vertical axis (y-axis) corresponds to −log 2 (*p*-value), and the horizontal axis (x-axis) displays the log 2-fold change (MetS-MSCs + Vit-C/MetS-MSCs). Peaks with higher (*n* = 25 peaks in 23 genes) and lower (*n* = 18 peaks in 17 genes) 5 hmC in MetS-MSCs + Vit-C versus MetS-MSCs are shown as red and blue dots, respectively, while non-significant peaks are shown as gray dots (*p*-value < 0.05 and fold changes ≥2 are indicated by black dashed lines). Heat map representing 25 peaks with higher (**B**) and 18 peaks with lower (**C**) 5 hmC levels in mitochondria-related genes of MetS-MSCs + Vit-C versus MetS-MSCs (*n* = 3 each). Functional annotation based on enrichment score of genes with high (**D**) or low (**E**) 5 hmC peaks in MetS-MSCs + Vit-C versus MetS-MSCs. (**F**) Representative 5 hmC profiles for the candidate mitochondrial genes reticulocalbin 2 (*RCN2*) and phorbol-12-myristate-13-acetate-induced protein 1 (*PMAIP1*) in MetS-MSCs + Vit-C and MetS-MSCs (Integrative Genomics Viewer). Gray rectangles indicate regions (peaks) with high (*RCN2*) and low (*PMAIP1*) 5 hmC. (**G**) Expression (qPCR) of the candidate mitochondrial genes cytochrome P450 family 11 subfamily A member 1 (*CYP11A1*) and methylmalonyl-CoA epimerase (*MCEE*) is consistent with hyper- and hypo-hydroxymethylated peaks, respectively, for these genes in MetS-MSCs + Vit-C versus MetS-MSCs (* *p* < 0.05 vs. Lean-MSCs) (*n* = 6 each).

Co-incubation of MetS-MSCs with Vit-C resulted in a total of 43 differentially hydroxymethylated regions (Figure 4A), including 25 high 5 hmC peaks corresponding to 23 genes (Figure 4B), and 18 low 5 hmC peaks corresponding to 17 genes (Figure 4C) in MetS-MSCs + Vit-C versus MetS-MSCs, of which only six high 5 hmC peaks (six genes) and nine Low 5 hmC peaks (nine genes) in MetS-MSCs compared to Lean-MSCs were reversed (changed direction) by Vit-C (Figure S4A,B). 

Functional annotation clustering analysis showed that mitochondria-related genes with high 5 hmC peaks in MetS-MSCs + Vit-C were primarily implicated in response to oxidative stress and antioxidant activity, including reactive oxygen species modulator 1 (*ROMO1*), peroxiredoxin 3 (*PRDX3*) and 5 (*PRDX5*), and cytochrome P450 family 11 subfamily A member 1 (*CYP11A1*), whereas genes with low 5 hmC peaks in MetS-MSCs + Vit-C were mostly involved in regulation of apoptosis, such as phorbol-12-myristate-13-acetate-induced protein 1 (*PMAIP1*) and methylmalonyl-CoA epimerase (*MCEE*) (Figure 4F,G). Expression of the candidate genes *CYP11A1* and *MCEE* followed the same patterns as the hMeDIP-seq findings, with higher and lower levels, respectively, in MetS-MSCs + Vit-C versus MetS-MSCs (Figure 4G). Co-incubation of Lean-MSCs with Vit-C resulted in a total of 28 differentially hydroxymethylated regions (Figure S5A), including 5 high 5 hmC peaks corresponding to 5 genes and 23 low 5 hmC peaks corresponding to 21 genes (Figure S5B) in Lean-MSCs + Vit-C versus Lean-MSCs. However, only four of these genes overlapped with hyper- or hypo-hydroxymethylated peaks in Vit-C-treated MetS-MSCs compared to untreated MetS-MSCs (Figure S5C).

Analysis of the overall 5 hmC landscape identified 210 peaks with higher and 210 with lower 5 hmC levels in MetS-MSCs + Vit-C versus MetS-MSCs, corresponding to 197 and 182 genes, respectively (Figure S6A). Epigenetic changes in mitochondrial genes (Figure 4) in MetS-MSCs accounted for 10.6% of all epigenetic changes induced by Vit-C (Figure S6B). Global 5 mC immunoreactivity was similar between Lean- and MetS-MSCs and remained unchanged in cells co-incubated with Vit-C (Figure S7). Expression of *TET1* and *TET2* was higher in MetS-MSCs compared to Lean-MSCs, but expression of TET3 was similar between the groups (Figure S8). 

### 3.6. Vit-C Attenuated Mitochondrial Structural Abnormalities and Dysfunction in MetS-MSCs 

**Figure 5:**
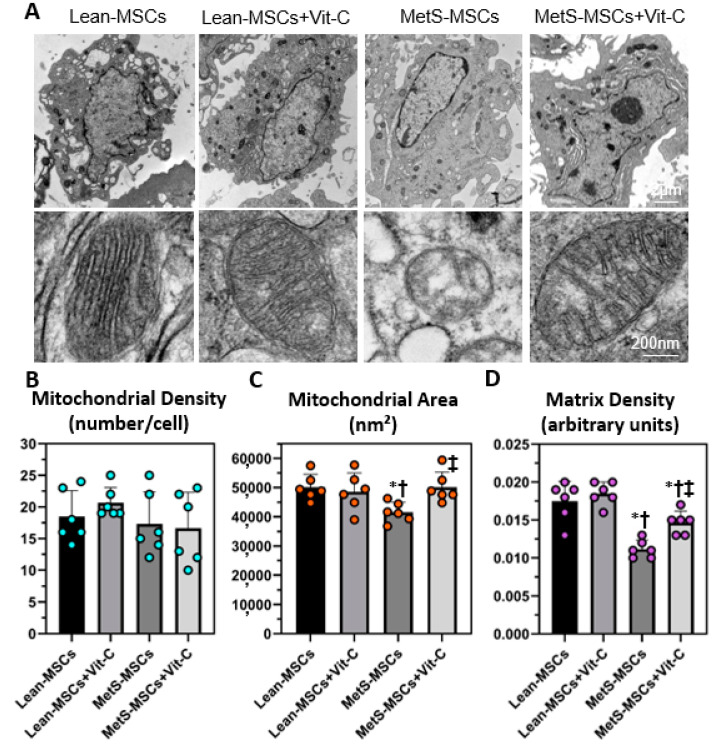
Vit-C attenuated mitochondrial structural abnormalities in MetS-MSCs. (**A**) Representative transmission electron microscopy images of Lean- and MetS-MSCs, untreated and treated with Vit-C (*n* = 6 each). Quantification of mitochondrial density (**B**), area (**C**), and matrix density (**D**) in all groups. * *p* < 0.05 vs. Lean-MSCs; † *p* < 0.05 vs. Lean + MSCs + Vit-C; ‡ *p* < 0.05 vs. MetS-MSCs.

**Figure 6:**
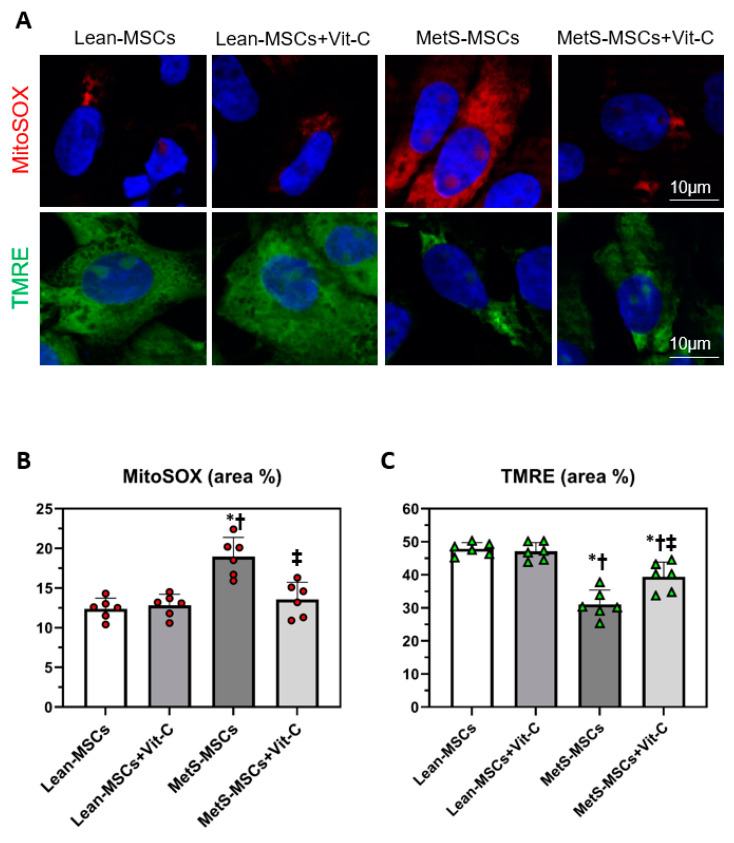
Vit-C improved mitochondrial function in MetS-MSCs. (**A**) Representative images of immunofluorescence (original magnification: X40) from triplicate stainings for the mitochondrial superoxide indicator MitoSOX (red) and the mitochondrial membrane potential marker tetramethylrhodamine ethylester (TMRE, green) of Lean- and MetS-MSCs, untreated and treated with Vit-C (*n* = 6 each). Quantification of mitochondrial reactive oxygen species (**B**) and membrane potential (**C**) in all groups. * *p* < 0.05 vs. Lean-MSCs; † *p* < 0.05 vs. Lean + MSCs + Vit-C; ‡ *p* < 0.05 vs. MetS-MSCs.

Mitochondrial density did not differ among the groups, whereas mitochondrial area and matrix density that decreased in MetS-MSCs compared to Lean groups improved in MetS-MSCs treated with Vit-C (Figure 5A–D). Mitochondrial production of superoxide (Mito-SOX) increased in MetS-MSCs compared to Lean-MSCs but was restored to Lean levels in MetS-MSCs treated with Vit-C (Figure 6A,B). Furthermore, mitochondrial membrane potential (TMRE), which decreased in MetS-MSCs compared to Lean-MSCs, improved in MetS-MSCs treated with Vit-C (Figure 6A,C), as did AMPK immunoreactivity (Figure S9). Co-incubation of MetS-MSCs with DMαKG restored expression of the candidate genes *HADHB, FASN, ETFB,* and *CYB5A* (Figure S10)*;* ameliorated mitochondrial oxidative stress (MitoSOX); and improved membrane potential (TMRE) (Figure S11).

## 4. Discussion

Mitochondria modulate several important aspects of MSC function, including plasticity, proliferation, and differential potential [46,47]. The current study found that MetS induces site-specific DNA hydroxymethylation (5 hmC) changes in nuclear-encoded mitochondrial genes in swine MSCs. Differentially methylated and hydroxymethylated regions have been previously described in placental [48], subcutaneous [49], and visceral [50] adipose tissue from patients with obesity. Platelet mitochondrial DNA methylation has been also reported to predict cardiovascular risk in obesity [51]. Our study extends these observations and suggest that epigenetic alterations in nuclear-encoded mitochondrial genes might represent a central pathogenic mechanism in obesity. 

It is the case that 5 hmC is increasingly gaining recognition as an stable epigenetic mark with high sensitivity to metabolic and inflammatory disease conditions [52], potential reversibility [53], capacity to bind to specific reader proteins [54], and positive association with gene transcription [55], in part through its participation in the active DNA demethylation pathway [56]. Previous studies reported that changes in 5 hmC levels may occur in diabetes [57], obesity [31], and hypertension [58], as well as preceding mitochondrial dysfunction in fatty-acid-treated cells [57]. In the current study, we identified 172 peaks with higher and 416 with lower 5 hmC levels in MetS- versus Lean-MSCs, which were annotated to 31 and 93 unique genes, respectively. Among the genes with hyper-hydroxymethylated peaks were *HADHA* and *HADHB*, which are involved in mitochondrial beta-oxidation of long chain fatty acids and cardiolipin metabolism [59,60], and translocator protein (*TSPO*), which is involved in cholesterol efflux and fatty acid oxidation [61,62]. Therefore, epigenetic changes in these genes may compromise various metabolic processes implicated in fatty acid metabolism.

Contrarily, genes with hypo-hydroxymethylated peaks were mainly implicated in oxidative phosphorylation and antioxidant activity, including cytochrome C oxidase subunit 6C (*COX6C*), which catalyzes the electron transfer from reduced cytochrome C to oxygen, and *UQCR10*, which encodes a subunit of mitochondrial complex III [63], as well as the antioxidant enzymes superoxide dismutase 1 (*SOD1*) and peroxiredoxin 6 (*PRDX6*), which catalyze the disproportionation of superoxide and reduction of hydrogen peroxide, respectively. Possibly, decreased activation of these genes in MetS-MSCs might interfere with diverse mitochondrial functions. Importantly, expression of randomly selected candidate genes (*HADHA*, *ALDH5A1*, *NDUFB2*, and *COX10*) matched their associated hyper- and hypo-hydroxymethylation patterns both in p-3 and p-0 MSCs, suggesting that MSCs have similar phenotypes after three passages. 

It is also important to note that the TET enzymes that catalyze oxidation of 5 mC to 5 hmC are members of a family of Fe^2+^ and α-ketoglutarate-dependent dioxygenases, competitive inhibitors that include the tricarboxylic acid cycle intermediates fumarate and succinate [64]. Catalytic activity of the TETs depends upon the function of succinate dehydrogenase, fumarate hydratase, and isocitrate dehydrogenase, as well as on ROS accumulation, which impinges on Fe^2+^ availability [53]. As a result, 5 hmC may be sensitive to mitochondrial impairment through retrograde signaling [65]. Therefore, MetS-induced changes in 5 hmC levels in mitochondria-related genes in MSCs may occur as a cause or a consequence of mitochondrial damage, creating a vicious cycle of epigenetic alterations and mitochondrial injury. In addition, we found that MetS altered the entire nuclear DNA genome of MSCs, including genes implicated in important cellular processes that may also contribute to mitochondrial damage, such as regulation of cell signaling and cell death [66]. 

To determine whether MetS-induced epigenetic changes in mitochondria-related genes impact on MSC mitochondrial structure and function, we treated cells with the epigenetic modulator Vit-C. The processes of 5 hmC generation and distribution are sensitive to alterations in the availability of co-factors for the TET enzymes, such as Vit-C [25], which enhances TET catalytic activity by promoting Fe^2+^ recycling, independently of its general antioxidant function [24]. In agreement, we found that expression of *TET1* and *TET2* was higher in MetS-MSCs compared to Lean-MSCs, which might be consistent with compensatory upregulation to mitigate possible depletion of major cofactors such as Vit-C. 

We identified 25 hyper- and 18 hypo-hydroxymethylated peaks in mitochondria-related genes in Vit-C-treated MetS-MSCs. Hyper-hydroxymethylated peaks were mapped to genes with antioxidant activity, including the *PRDX3* and *PRDX5*, which regulate mitochondrial hydrogen peroxide levels [67]. Previous studies have shown that *PRDX3* is downregulated in human adipocytes, which contributes to oxidative stress and defective mitochondrial biogenesis [68], suggesting a potential benefit from its activation. Vit-C can also scavenge reactive oxygen species and undergo oxidation to dehydroascorbic acid, so its bioavailability in the nucleus as a TET enzyme co-factor is regulated by the redox status of the cell [69]. Therefore, Vit-C-induced epigenetic activation of antioxidant genes may generate positive feedback to reduce mitochondrial and cellular oxidative stress. 

Although Vit-C promotes TET catalytic activity and 5 hmC formation [24], it also acts as a co-factor to enhance and maintain the activity of many other α-ketoglutarate-dependent dioxygenases [70] and has been associated with bi-directional effects on gene expression [71]. In line with this, we found that co-incubation of MetS-MSCs with Vit-C induced hypo-hydroxymethion in genes implicated in regulation of apoptosis, including the *NDUFA4* mitochondrial complex associated (*NDUFA4*), which inhibits apoptosis through B-cell lymphoma 2 and the cytochrome-C-mediated signaling pathway [72]. We have previously shown that swine MetS-MSCs have propensity for senescence [73], which unlike apoptosis is not pre-determined and results in the secretion of cytokines and inflammatory mediators (senescence-associated secretory phenotype). Speculatively, epigenetic inactivation of anti-apoptotic genes by Vit-C may partly play a role in modulating early senescence in MetS-MSCs. 

Hyper- and hypo-hydroxymethylated peaks in mitochondria-related genes were also observed in a small number of Vit-C-treated Lean-MSCs compared to untreated Lean-MSCs. However, only a few genes exhibited hyper- or hypo-hydroxymethylated peaks in Vit-C-treated MetS-MSCs compared to untreated MetS-MSCs, suggesting that Vit-C exerts less prominent and distinct epigenetic modulation in Lean- compared MetS-MSCs. Global 5 mC immunoreactivity was similar between Lean- and MetS-MSCs and remained unchanged in cells co-incubated with Vit-C, arguing against a major role of MetS and Vit-C in modulating global methylation levels in MSCs. Epigenetic changes in nuclear-encoded mitochondrial genes in MetS-MSCs accounted for a relatively small percentage of all epigenetic changes induced by Vit-C, suggesting that Vit-C might be also implicated in regulating other cellular processes. Collectively, these results underscore the ability of Vit-C to modulate epigenetic changes in genes implicated in oxidative stress and apoptosis, consistent with previous reports of the effect of DNA methyltransferase inhibitors on equine [74] and human [75] adipose-derived MSCs.

Mitochondrial fragmentation (restoration of mitochondrial area) and cristae remodeling and loss (improvement of matrix density) were attenuated in MetS-MSCs co-incubated with Vit-C. Mitochondria contributes to several aspects of cellular metabolism, in part by generating biosynthetic precursors for macromolecules and maintaining redox homeostasis [76]. Here, we found that co-incubation with either Vit-C or DMαKG ameliorated mitochondrial superoxide production and improved mitochondrial membrane potential, the driving force for mitochondrial ATP synthesis. Furthermore, Vit-C restored AMPK levels, underscoring the potential of this epigenetic co-factor to ameliorate MetS-induced MSC metabolic perturbations. Therefore, these observations suggest that epigenetic changes might partly account for impaired mitochondrial metabolic state in MetS-MSCs and position Vit-C and DMαKG as a potential therapeutic option to ameliorate the deleterious effects of MetS on MSC mitochondria. However, systemic administration of Vit-C may exert pro-oxidant effects [77,78] and poor efficacy in clinical trials [79]. Therefore, the use of Vit-C as a preconditioning method for improving the efficiency of MSCs before autologous transplantation would be much preferable than its systemic administration.

We acknowledge limitations in our study, such as the use of adipose tissue MSCs harvested from relatively young animals with early stages of MetS. Nevertheless, our diet-induced large animal model closely mimics the main characteristics of patients with MetS. The number of samples used for hMeDIP-seq was modest, as often used in seq studies [33,34], due to the costs associated with these approaches. Yet, this sample size clearly sufficed to detect significant differences in 5 hmC levels between Lean- and MetS-MSCs. Although increased rate of duplicates and immunoprecipitation bias in hMeDIP-seq may result in data loss and potentially false positives [80], we mitigated this effect by qPCR studies, which confirmed the direction and significance of several epigenetic changes in MetS-MSCs. Lastly, our MSC culture media contained a negligible amount of Vit-C (2.5 mg/L); however, given that Lean- and MetS-MSCs were cultured in a similar way, differences observed in 5 hmC levels clearly reflect the effect of MetS on mitochondria-related genes of swine MSCs.

## 5. Conclusions

In summary, we characterized and compared the 5 hmC landscape of mitochondria-related genes in swine Lean- and MetS-MSCs and found that nuclear-encoded mitochondrial genes with differential 5 hmC peaks were primarily implicated in fatty acid metabolism and electron transport chain activity. Although Vit-C only reversed 5 hmC levels of few genes, it increased 5 hmC levels in mitochondrial antioxidant genes and attenuated mitochondrial ROS generation, structural abnormalities, and dysfunction. Therefore, our observations may contribute to development of strategies to enhance the reparative capacity of MSCs in individuals suffering from MetS. Further studies are needed to confirm and support these findings and evaluate the possibility of epigenetic reversal/restoration using Vit-C or other epigenetic modulators in human MSCs.

## Figures and Tables

**Table 1:** Systemic characteristics in experimental groups (*n* = 6, each) at 16 weeks.

Parameter	Lean	MetS
Body weight (Kg)	74.4 ± 10.2	91.4 ± 2.3 *
Mean blood pressure (mmHg)	98.4 ± 11.0	129.5 ± 8.2 *
Total cholesterol (mg/dL)	82.7 ± 6.2	469.9 ± 59.4 *
LDL cholesterol (mg/dL)	33.3 ± 6.1	350.1 ± 126.3 *
Triglycerides (mg/dL)	8.0 ± 1.2	21.4 ± 5.5 *
Fasting glucose (mg/dL)	120.9 ± 11.9	117.1 ± 13.3
Fasting insulin (µU/mL)	0.4 ± 0.1	0.7 ± 0.1 *
HOMA-IR score	0.7 ± 0.1	1.9 ± 0.1 *

* *p* < 0.05 vs. Lean. MetS: metabolic syndrome, LDL: low-density lipoprotein, HOMA-IR: homeostasis model assessment of insulin resistance.

## Data Availability

The raw hMeDIP-seq data for this paper are available at repository name: Zenodo. Data identification number: doi:10.5281/zenodo.7789769.

## Supplementary Materials

The following supporting information can be downloaded at https://www.mdpi.com/article/10.3390/cells12091274/s1. Supplementary Figures S1–S11 and their respective legends are available at repository name: Zenodo Data identification number: (accessed on 16 March 2023).
