# Supplementary material for: Metabolic Syndrome Induces Epigenetic Alterations in Mitochondria-Related Genes in Swine Mesenchymal Stem Cells
Source: Cells. 2023 Apr 27;12(9):1274. doi: 10.3390/cells12091274 (PMC10177475; doi:10.3390/cells12091274)
Supplement: Supplementary file 1 [file cells-12-01274-s001.zip › cells-2212076-supplementary.pdf]

Supplemental Figure Legends

Figure S1. *Interactions among genes with high 5hmC peaks in MetS-MSCs*. (**a**): Clusters of physical and functional protein interactions among mitochondria-related genes with high 5hmC peaks in MetS-MSCs (higher strength of interaction represented by multiple strings between the genes shown in different colors). (**b**): Expression (qPCR) of the candidate mitochondrial genes Hydroxyacyl-CoA Dehydrogenase Trifunctional Multienzyme Complex Subunit Alpha (*HADHA*) and Aldehyde Dehydrogenase 5 Family Member A1 (*ALDH5A1*) was higher in passage (p)-0 MetS-MSCs versus p-0 Lean-MSCs.

Figure S2. *Interactions among genes with low 5hmC peaks in MetS-MSCs*. (**a**): Clusters of physical and functional protein interactions among mitochondria-related genes with low 5hmC peaks in MetS-MSCs (higher strength of interaction represented by multiple strings between the genes shown in different colors). (**b**): Expression (qPCR) of mitochondrial genes NADH: Ubiquinone Oxidoreductase Subunit B6 (*NDUFB2*) and Cytochrome C Oxidase Assembly Factor Heme A Farnesyltransferase COX10 (*COX10*) was lower in p-0 MetS-MSCs versus p-0 Lean-MSCs. (*p<0.05 vs. Lean-MSCs).

Figure S3. *MetS alters the entire 5hmC profile of swine adipose tissue derived MSCs*. (**a**): Volcano plot showing 317,733 5-hydroxymethylcytosine (5hmC) peaks in genes, of which 7,022 were higher and 9,733 lower in MetS-MSCs compared to Lean-MSCs. The vertical axis (y-axis) corresponds to -log 2 (p-value) and the horizontal axis (x-axis) displays log 2-fold change (MetS-/Lean-MSCs). Higher (n=7,022 peaks in 2,142 genes) and lower (n=9,733 peaks in 2,604 genes) 5hmC peaks in MetS- versus Lean-MSCs are shown as red and blue dots, respectively, while non-significant peaks are shown as gray dots (p-value <0.05 and fold changes ≥2 are indicated by black dashed lines). (**b**): Gene set enrichment analysis (GSEA) of genes with high 5hmC peaks in MetS-MSCs versus Lean-MSCs (**c**): GSEA of genes with low 5hmC peaks in MetS-MSCs versus Lean-MSCs.

Figure S4. *Vitamin (Vit)-C reversed few high and low 5hmC peaks in MetS-MSCs*. (**a**): Volcano plot showing that among 172 high 5hmC peaks in mitochondria-related genes in MetS-MSCs compared to Lean-MSCs, 6 (corresponding to 6 genes) were reversed in MetS-MSCs treated with Vit-C (50μg/mL for 48hrs). The vertical axis (y-axis) corresponds to -log 2 (p-value) and the horizontal axis (x-axis) displays the log 2-fold change (MetS-MSCs+Vit-C/MetS-MSCs). Peaks with lower (n=6 peaks in 6 genes) 5hmC in MetS-MSCs+Vit-C versus MetS-MSCs are shown in blue dots, while non-significant or high peaks are shown as gray dots. (**b**): Volcano plot showing that among 416 low 5hmC peaks in mitochondria-related genes in MetS-MSCs compared to Lean-MSCs, 9 (corresponding to 9 genes) were reversed in MetS-MSCs treated with Vit-C (50μg/mL for 48hrs). The vertical axis (y-axis) corresponds to -log 2 (p-value) and the horizontal axis (x-axis) displays the log 2-fold change (MetS-MSCs+Vit-C/MetS-MSCs). Peaks with higher (n=9 peaks in 9 genes) 5hmC in MetS-MSCs+Vit-C versus MetS-MSCs are shown in red dots, while non-significant or high peaks are shown as gray dots.

Figure S5. *Vitamin (Vit)-C modulates 5hmC levels in mitochondria-related genes in Lean-MSCs*. (**a**): Volcano plot showing 23,944 5hmC peaks in mitochondria-related genes identified in Lean-MSCs treated with Vit-C (50μg/mL for 48hrs) versus Lean-MSCs untreated (Lean-MSCs+Vit-C/Lean-MSCs). The vertical axis (y-axis) corresponds to -log 2 (p-value) and the horizontal axis (x-axis) displays the log 2-fold change (Lean-MSCs+Vit-C/Lean-MSCs). Peaks with higher (n=5 peaks in 5 genes) and lower (n=23 peaks in 21 genes) 5hmC in Lean-MSCs+Vit-C versus Lean-MSCs are shown as red and blue dots, respectively, while non-significant peaks are shown as gray dots (p-value <0.05 and fold changes ≥2 are indicated by black dashed lines) (**b**): Heat map representing 5 peaks in mitochondria-related genes with higher (left) and lower (right) 5hmC levels in Lean-MSCs+Vit-C versus Lean-MSCs (n=3 each). (**c**): Venn diagram showing that only 4 of these genes also exhibited hyper or hypo- hydroxymethylated peaks in Vit-C-treated MetS-MSCs compared to untreated MetS-MSCs.

Figure S6. *Vitamin (Vit)-C modulates 5hmC levels in swine MetS-MSC genes*. (**a**): Volcano plot showing 301,647 5hmC peaks in MetS-MSCs treated with Vit-C (50μg/mL for 48hrs) versus MSCs untreated (MetS-MSCs+Vit-C/MetS-MSCs). The vertical axis (y-axis) corresponds to -log 2 (p-value) and the horizontal axis (x-axis) displays the log 2-fold change (MetS-MSCs+Vit-C/MetS-MSCs). Peaks with higher (n=210 peaks in 197 genes) and lower (n=210 peaks in 182 genes) 5hmC in MetS-MSCs+Vit-C versus MetS-MSCs are shown as red and blue dots, respectively, while non-significant peaks are shown as gray dots (p-value <0.05 and fold changes ≥2 are indicated by black dashed lines) (n=3 each) (**b**):

Pie chart showing that epigenetic changes in mitochondrial genes accounted for 10.6% of all epigenetic changes induced by Vit-C.

Figure S7. *Global 5mC levels are similar among the groups*. (**a**): Representative images of immunofluorescence (original magnification: X40) for 5mC (red) and its quantification in Lean- and MetS-MSCs untreated and treated with Vit-C (n=6 each).

Figure S8. *MetS increases expression of epigenetic enzymes in swine MSCs*. Gene expression (qPCR) of the epigenetic enzymes Ten-Eleven Translocation (TET) methylcytosine dioxygenases 1 , 2, and 3 in MetS- compared to Lean-MSCs (n=6 each) (*p<0.05 vs. Lean-MSCs).

Figure S9. *Vitamin (Vit)-C restored AMPK immunoreactivity in MetS-MSCs*. (**a**): Immunofluorescence staining for 5' AMP-activated protein kinase (AMPK) and its quantification in Lean- and MetS-MSCs untreated or treated with Vit-C (n=6 each) (*p<0.05 vs. Lean-MSCs, †p<0.05 vs. Lean-MSCs+Vit-C, ‡p<0.05 vs. MetS-MSCs).

Figure S10. *Dimethyl alpha-ketoglutarate (DMαKG)*. Expression (qPCR) of mitochondrial genes *HADHB*: Hydroxyacyl-CoA Dehydrogenase Trifunctional Multienzyme Complex Subunit Beta, *FASN*: Fatty Acid Synthase, *ETFB*: Electron Transfer Flavoprotein Subunit Beta, and *CYB5A*: Cytochrome B5 Type A in Lean-MSCs and MetS-MSCs untreated or treated with *DMαKG*. (*p<0.05 vs. Lean-MSCs). *p<0.05 vs. Lean-MSCs; †p<0.05 vs. Lean+MSCs+DMαKG; ‡p<0.05 vs. MetS-MSCs.

Figure S11. *DMαKG ameliorates oxidative stress and improves membrane potential in MetS-MSCs*. (**a**): Representative images of immunofluorescence (original magnification: X40) for the mitochondrial superoxide indicator MitoSOX (red) and the mitochondrial membrane potential marker tetramethylrhodamine ethylester (TMRE, green) of Lean- and MetS-MSCs, untreated and treated with DMαKG (n=6 each). Quantification of mitochondrial reactive oxygen species (**b**) and membrane potential (**c**) in all groups. *p<0.05 vs. Lean-MSCs; †p<0.05 vs. Lean+MSCs+DMαKG; ‡p<0.05 vs. MetS-MSCs.

Figure S1

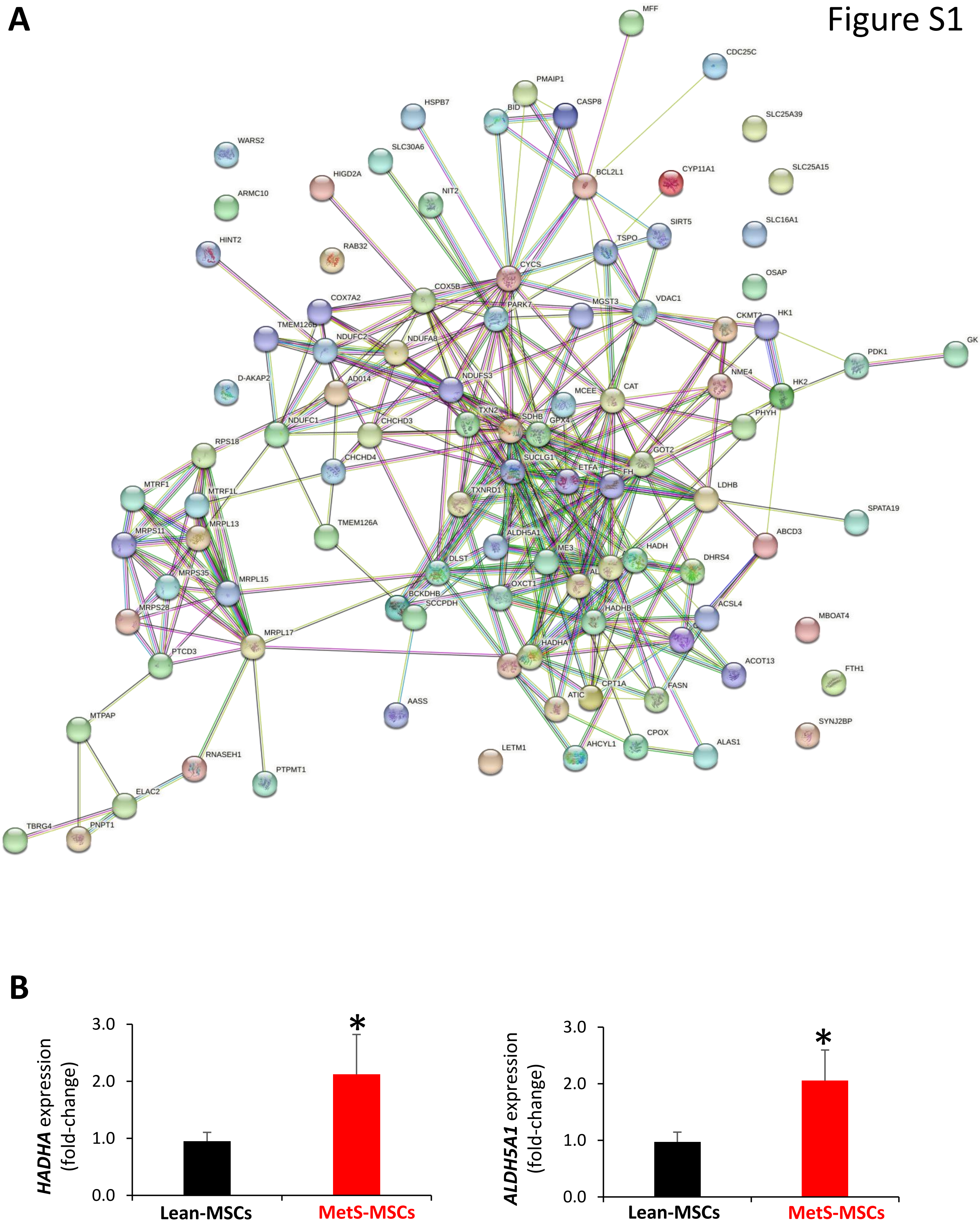

A
MFF
CDC25C
PMAIP1
CASP8
BID
HSPB7
SLC25A39
WARS2
SLC30A6
HIGD2A
BCL2L1
CYP11A1
SLC25A15
ARMC10
NIT2
SIRT5
SLC16A1
HINT2
RAB32
TSPO
CYCS
OSAP
COX5B
COX7A2
PARK7
MGST3
VDAC1
CKMT2
HK1
GK
TMEM126B
NDUFC2
NDUFA8
PDK1
D-AKAP2
AD014
NDUFS3
NME4
HK2
MCEE
CAT
TXN2
SDHB
GPX4
PHYH
NDUFC1
CHCHD3
RPS18
GOT2
SUCLG1
CHCHD4
ETFA
FH
LDHB
MTRF1
MTRF1L
TXNRD1
SPATA19
MRPL13
TMEM126A
MRPS11
ABCD3
ALDH5A1
HADH
ME3
DHRS4
MRPS35
DLST
MRPL15
OXCT1
BCKDHB
ACSL4
MBOAT4
MRPS28
SCCPDH
HADHB
MRPL17
HADHA
PTCD3
ACOT13
FTH1
CPT1A
FASN
ATIC
AASS
MTPAP
SYNJ2BP
CPOX
AHCYL1
ALAS1
LETM1
RNASEH1
PTPMT1
ELAC2
TBRG4
PNPT1
B
HADHA expression (fold-change)
ALDH5A1 expression (fold-change)
3.0
2.0
1.0
0.0
*
Lean-MSCs
MetS-MSCs

Figure S2

A

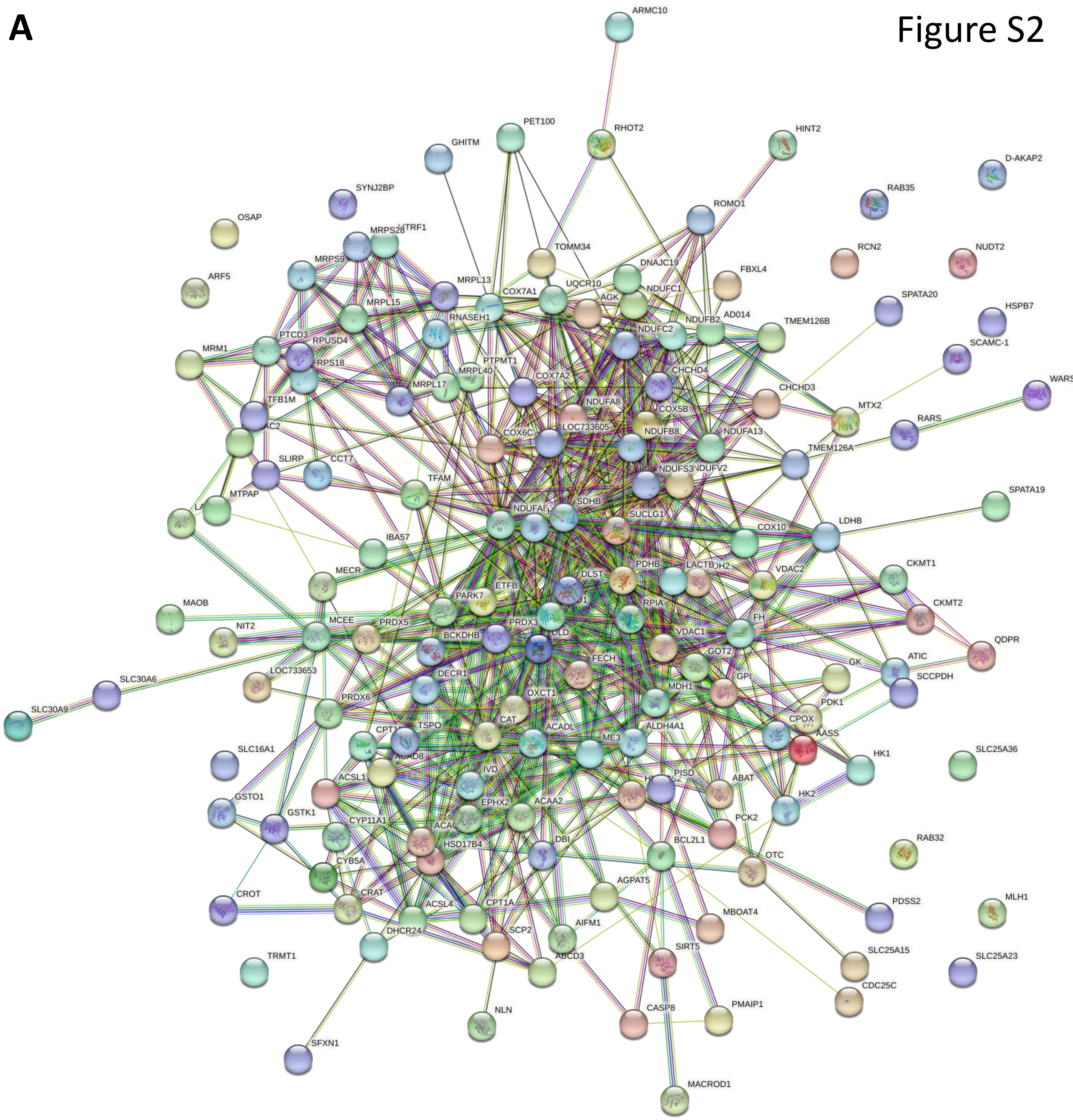
ARMC10
PET100
RHOT2
HINT2
GHITM
D-AKAP2
SYNJ2BP
RAB35
ROMO1
OSAP
MRPS28
HTRF1
TOMM34
RCN2
NUDT2
ARF5
MRPS9
DNAJC19
FBXL4
MRPL13
COX7A1
UQCR10
NDUFC1
MRPL15
AGK
SPATA20
HSPB7
RNASEH1
NDUFB2
AD014
TMEM126B
SCAMC-1
PTCD3
RPUSD4
NDUFC2
MRM1
RPS18
PTPMT1
MRPL40
CHCHD4
COX7A2
MRPL17
CHCHD3
TFB1M
NDUFA8
COX5B
MTX2
RARS
COX6C
LOC733605
NDUFB8
NDUFA13
SLIRP
CCT7
TMEM126A
TFAM
NDUFS3
MTPAP
SPATA19
SDHB
SUCLG1
LDHB
COX10
IBA57
CKMT1
PDHB
MECR
VDAC2
DLST
ETFB
PARK7
CKMT2
MAOB
RPIA
MCEE
PRDX5
FH
QDPR
NIT2
PRDX3
BCKDHB
DLD
VDAC1
GOT2
GK
ATIC
FECH
SLC30A6
LOC733653
SCCPDH
DECR1
GPI
MDH1
PDK1
SLC30A9
PRDX6
OXCT1
CPOX
CAT
TSPO
ACADL
ALDH4A1
AASS
ME3
SLC16A1
HK1
SLC25A36
ACSL1
IVD
PISD
ABAT
GSTO1
HK2
EPHX2
ACAA2
GSTK1
CYP11A1
PCK2
RAB32
HSD17B4
DBI
BCL2L1
OTC
CYB5A
CROT
CRAT
AGPAT5
PDSS2
MLH1
ACSL4
CPT1A
MBOAT4
DHCR24
SCP2
AIFM1
TRMT1
ABCD3
SIRT5
SLC25A15
SLC25A23
CDC25C
NLN
CASP8
PMAIP1
SFXN1
MACROD1

B

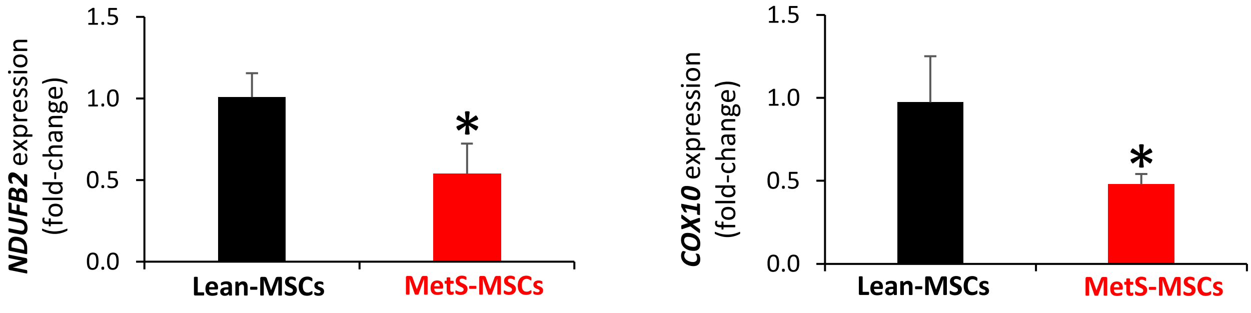
1.5
1.0
0.5
0.0
NDUFB2 expression (fold-change)
*
Lean-MSCs
MetS-MSCs
1.5
1.0
0.5
0.0
COX10 expression (fold-change)
*
Lean-MSCs
MetS-MSCs

Figure S3

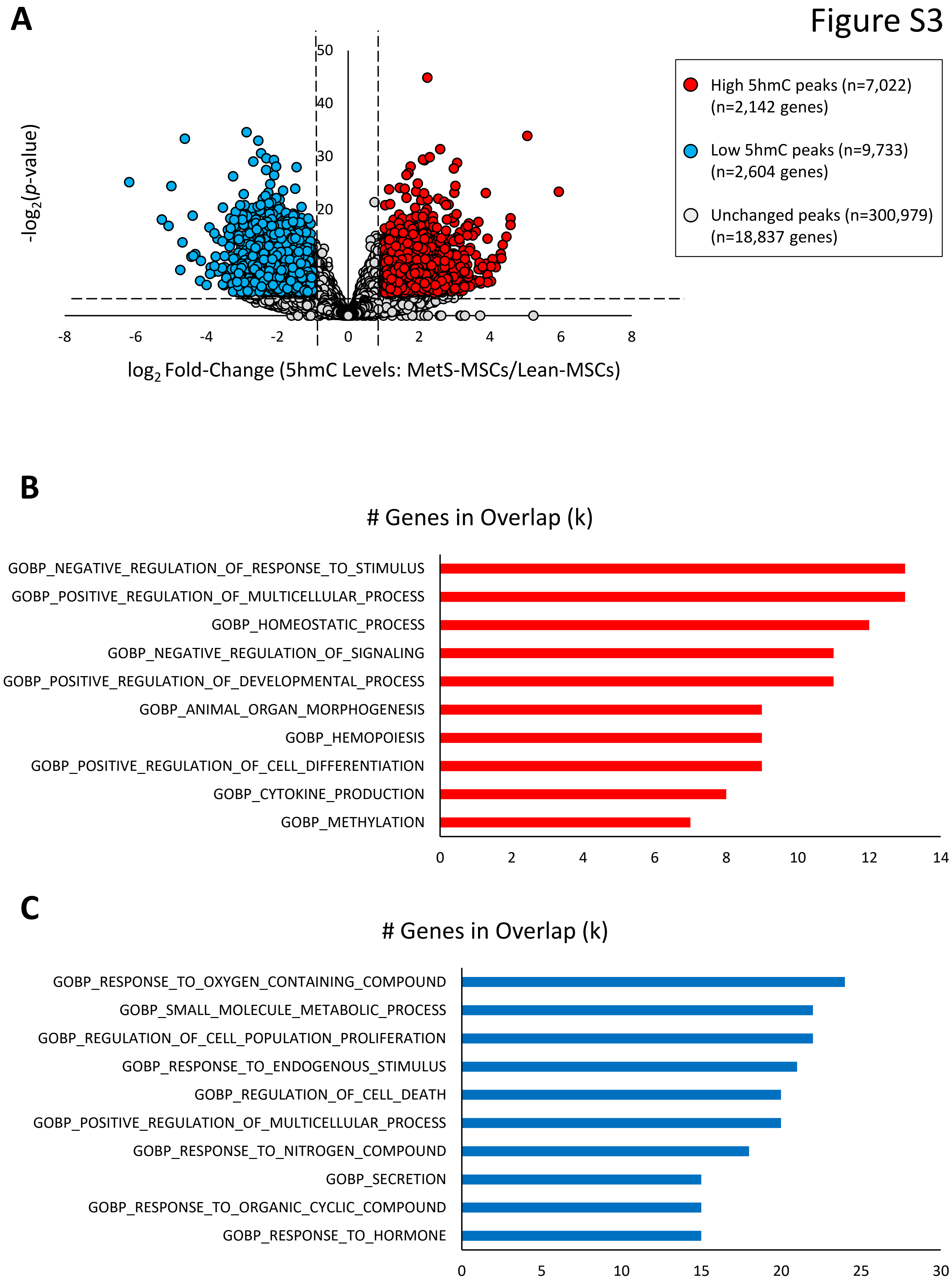
A
-log2(p-value)
50
40
30
20
-8
-6
-4
-2
0
2
4
6
8
log2 Fold-Change (5hmC Levels: MetS-MSCs/Lean-MSCs)
High 5hmC peaks (n=7,022)
(n=2,142 genes)
Low 5hmC peaks (n=9,733)
(n=2,604 genes)
Unchanged peaks (n=300,979)
(n=18,837 genes)
B
Genes in Overlap (k)
GOBP_NEGATIVE_REGULATION_OF_RESPONSE_TO_STIMULUS
GOBP_POSITIVE_REGULATION_OF_MULTICELLULAR_PROCESS
GOBP_HOMEOSTATIC_PROCESS
GOBP_NEGATIVE_REGULATION_OF_SIGNALING
GOBP_POSITIVE_REGULATION_OF_DEVELOPMENTAL_PROCESS
GOBP_ANIMAL_ORGAN_MORPHOGENESIS
GOBP_HEMOPOIESIS
GOBP_POSITIVE_REGULATION_OF_CELL_DIFFERENTIATION
GOBP_CYTOKINE_PRODUCTION
GOBP_METHYLATION
0
2
4
6
8
10
12
14
C
Genes in Overlap (k)
GOBP_RESPONSE_TO_OXYGEN_CONTAINING_COMPOUND
GOBP_SMALL_MOLECULE_METABOLIC_PROCESS
GOBP_REGULATION_OF_CELL_POPULATION_PROLIFERATION
GOBP_RESPONSE_TO_ENDOGENOUS_STIMULUS
GOBP_REGULATION_OF_CELL_DEATH
GOBP_POSITIVE_REGULATION_OF_MULTICELLULAR_PROCESS
GOBP_RESPONSE_TO_NITROGEN_COMPOUND
GOBP_SECRETION
GOBP_RESPONSE_TO_ORGANIC_CYCLIC_COMPOUND
GOBP_RESPONSE_TO_HORMONE
0
5
10
15
20
25
30

Figure S4

A

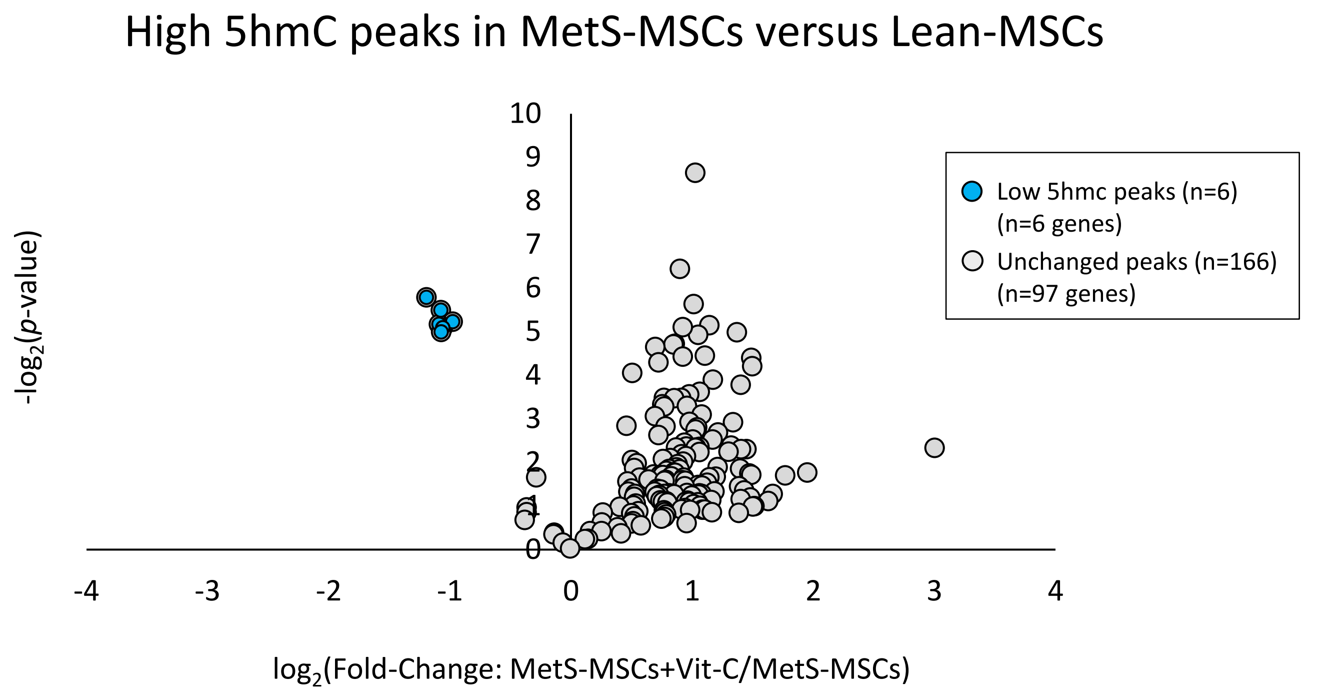
High 5hmC peaks in MetS-MSCs versus Lean-MSCs
-log2(p-value)
Low 5hmc peaks (n=6)
(n=6 genes)
Unchanged peaks (n=166)
(n=97 genes)
log2(Fold-Change: MetS-MSCs+Vit-C/MetS-MSCs)

B

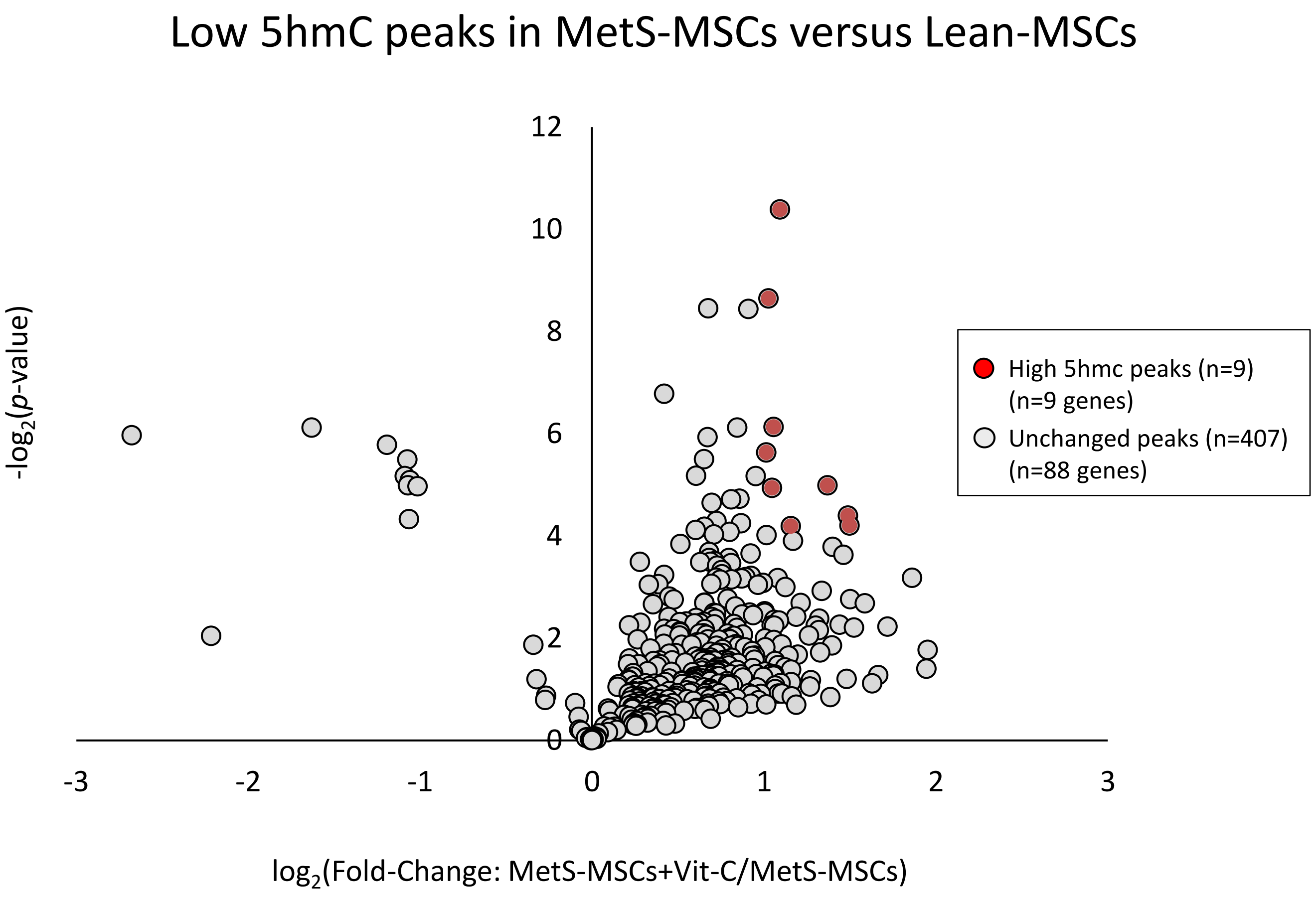
Low 5hmC peaks in MetS-MSCs versus Lean-MSCs
-log2(p-value)
High 5hmc peaks (n=9)
(n=9 genes)
Unchanged peaks (n=407)
(n=88 genes)
log2(Fold-Change: MetS-MSCs+Vit-C/MetS-MSCs)

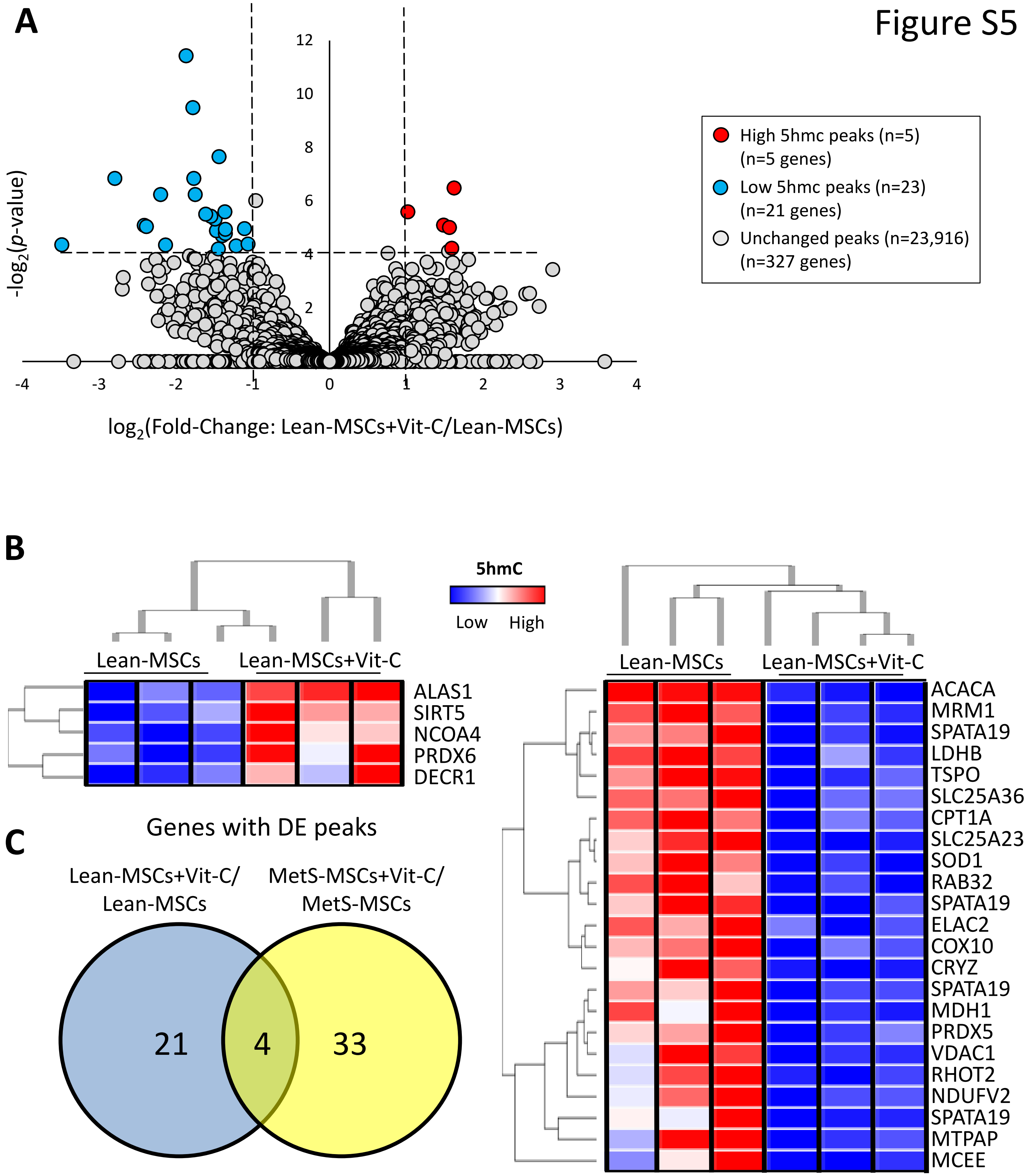

Figure S5
A
12
10
8
6
4
2
-4
-3
-2
-1
0
1
2
3
4
-log2(p-value)
log2(Fold-Change: Lean-MSCs+Vit-C/Lean-MSCs)
High 5hmc peaks (n=5)
(n=5 genes)
Low 5hmc peaks (n=23)
(n=21 genes)
Unchanged peaks (n=23,916)
(n=327 genes)
B
5hmC
Low
High
Lean-MSCs
Lean-MSCs+Vit-C
ALAS1
SIRT5
NCOA4
PRDX6
DECR1
Lean-MSCs
Lean-MSCs+Vit-C
ACACA
MRM1
SPATA19
LDHB
TSPO
SLC25A36
CPT1A
SLC25A23
SOD1
RAB32
SPATA19
ELAC2
COX10
CRYZ
SPATA19
MDH1
PRDX5
VDAC1
RHOT2
NDUFV2
SPATA19
MTPAP
MCEE
C
Genes with DE peaks
Lean-MSCs+Vit-C/
Lean-MSCs
MetS-MSCs+Vit-C/
MetS-MSCs
21
4
33

A

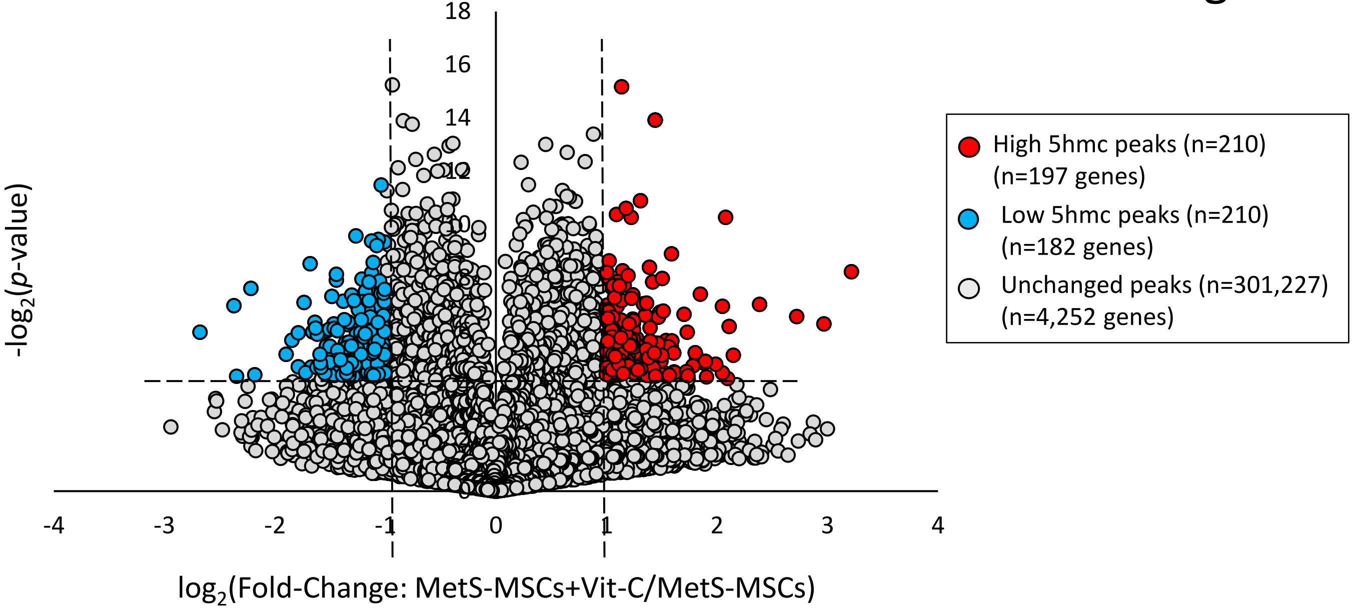

18
16
14
12
10
-log2(p-value)
-4
-3
-2
-1
0
1
2
3
4
log2(Fold-Change: MetS-MSCs+Vit-C/MetS-MSCs)
High 5hmc peaks (n=210)
(n=197 genes)
Low 5hmc peaks (n=210)
(n=182 genes)
Unchanged peaks (n=301,227)
(n=4,252 genes)

B

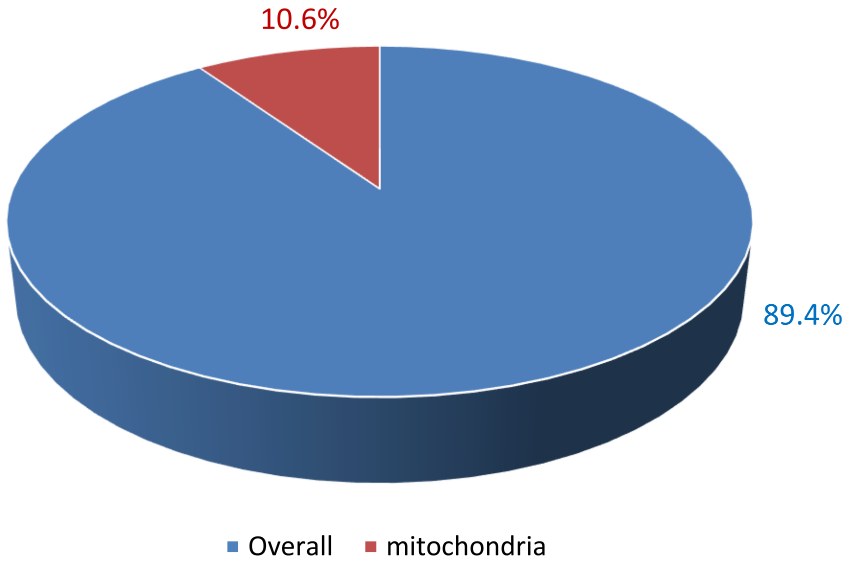

10.6%
89.4%
Overall
mitochondria

Figure S7

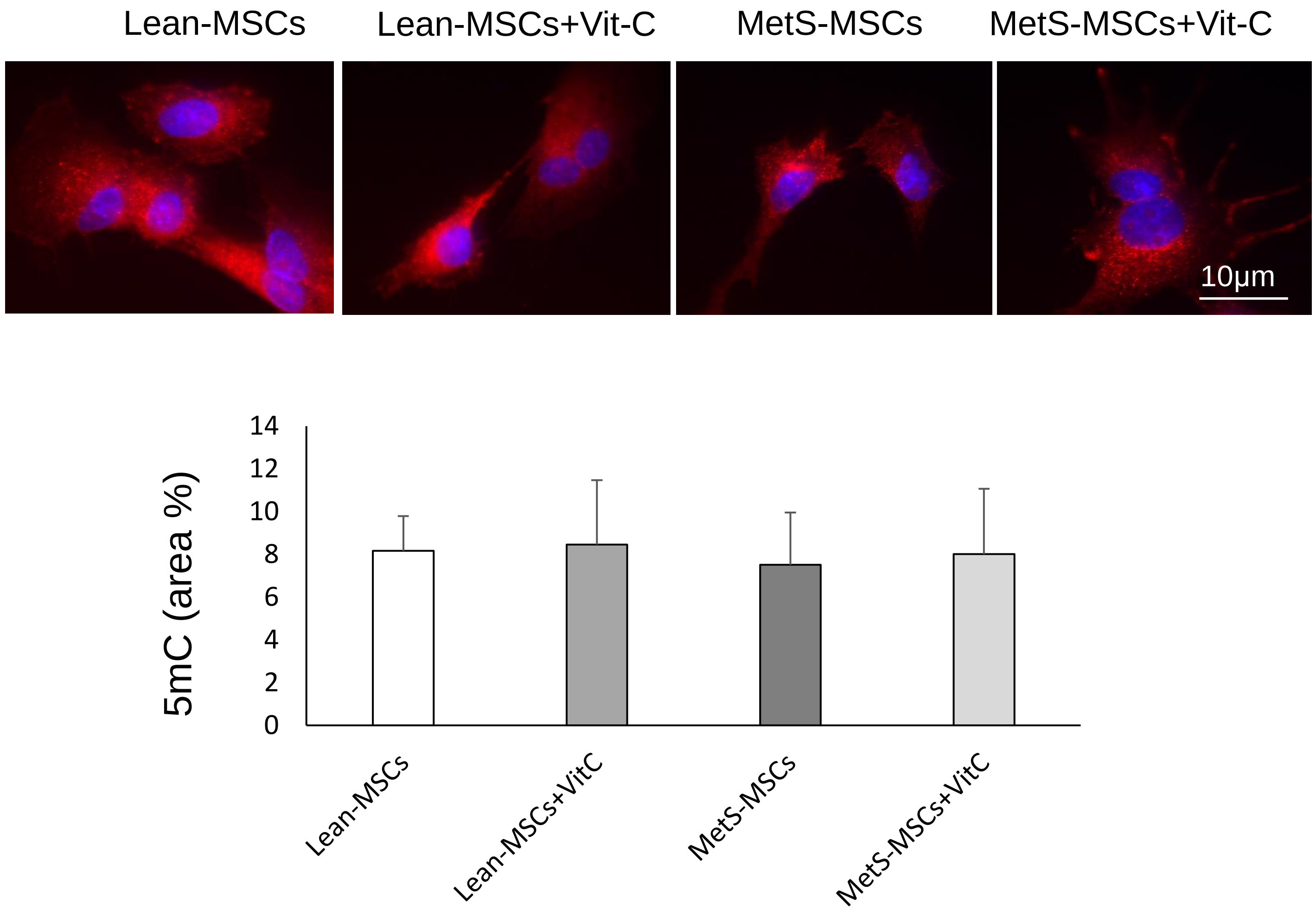
Lean-MSCs
Lean-MSCs+Vit-C
MetS-MSCs
MetS-MSCs+Vit-C
10μm
5mC (area %)
14
12
10
8
6
4
2
0
Lean-MSCs
Lean-MSCs+VitC
MetS-MSCs
MetS-MSCs+VitC

Figure S8

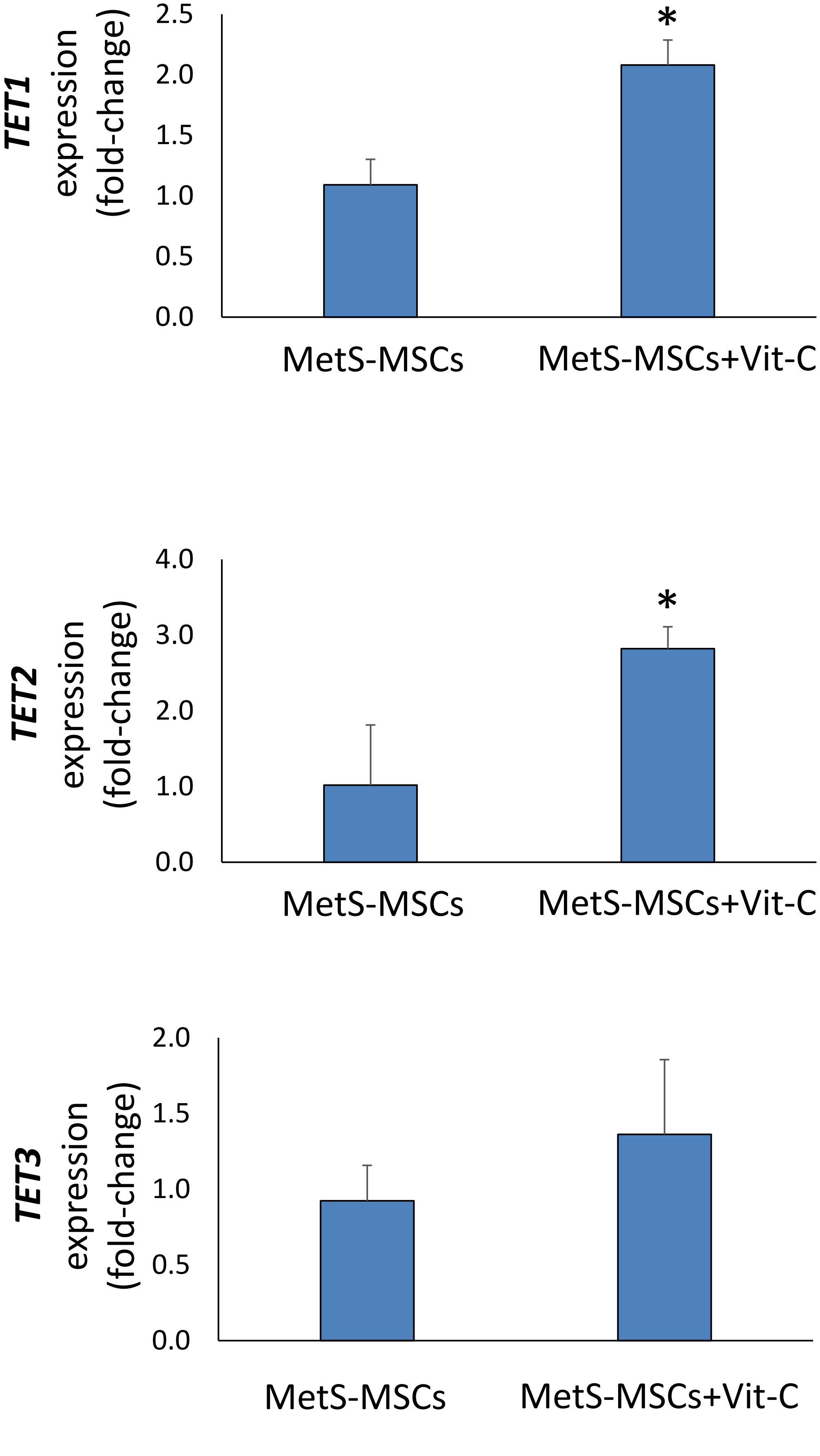
TET1
expression
(fold-change)
2.5
2.0
1.5
1.0
0.5
0.0
*
MetS-MSCs
MetS-MSCs+Vit-C
TET2
expression
(fold-change)
4.0
3.0
2.0
1.0
0.0
*
MetS-MSCs
MetS-MSCs+Vit-C
TET3
expression
(fold-change)
2.0
1.5
1.0
0.5
0.0
MetS-MSCs
MetS-MSCs+Vit-C

Figure S9

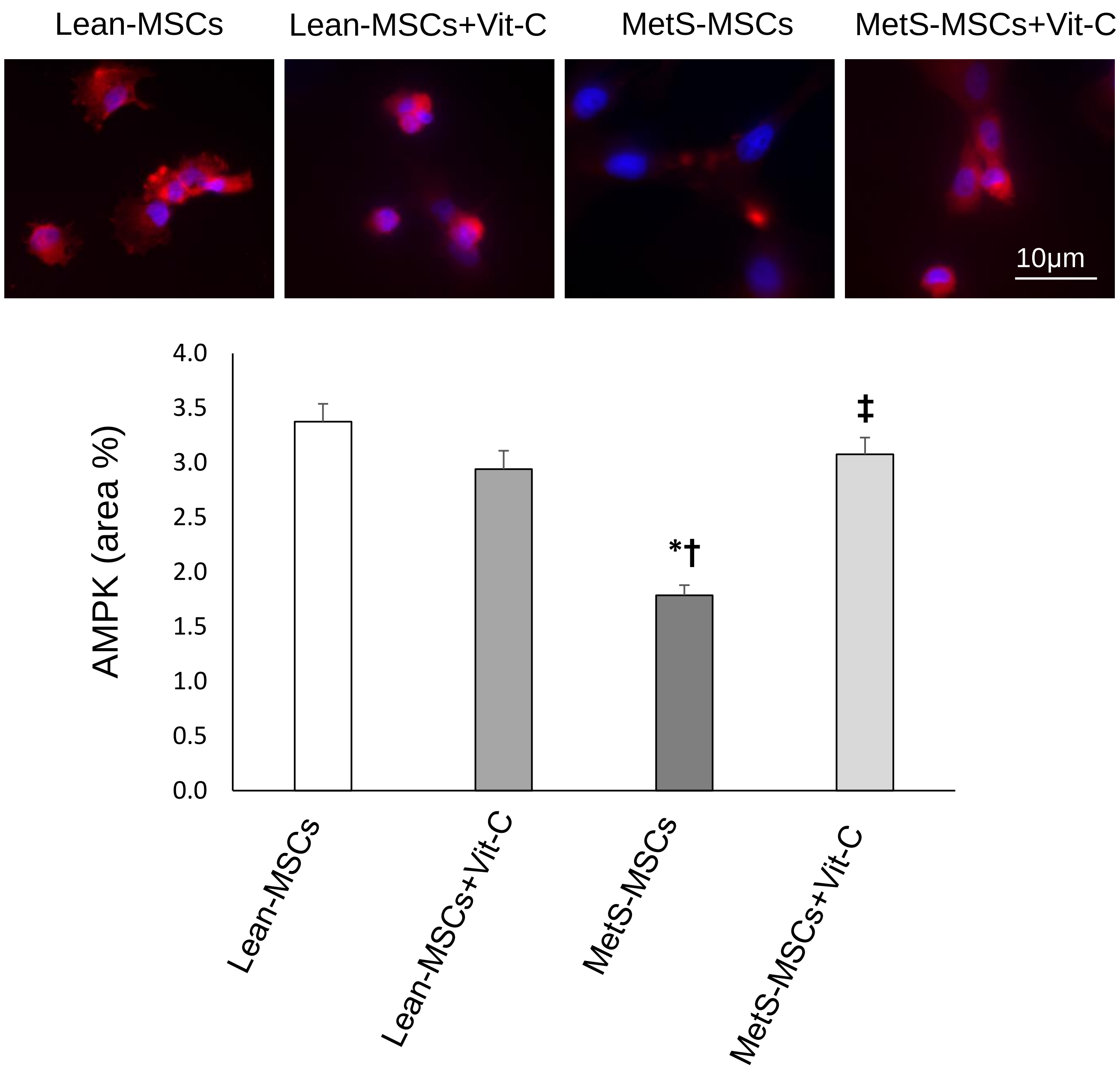
Lean-MSCs
Lean-MSCs+Vit-C
MetS-MSCs
MetS-MSCs+Vit-C
10μm
AMPK (area %)
4.0
3.5
3.0
2.5
2.0
1.5
1.0
0.5
0.0
*†
‡
Lean-MSCs
Lean-MSCs+Vit-C
MetS-MSCs
MetS-MSCs+Vit-C

Figure S10

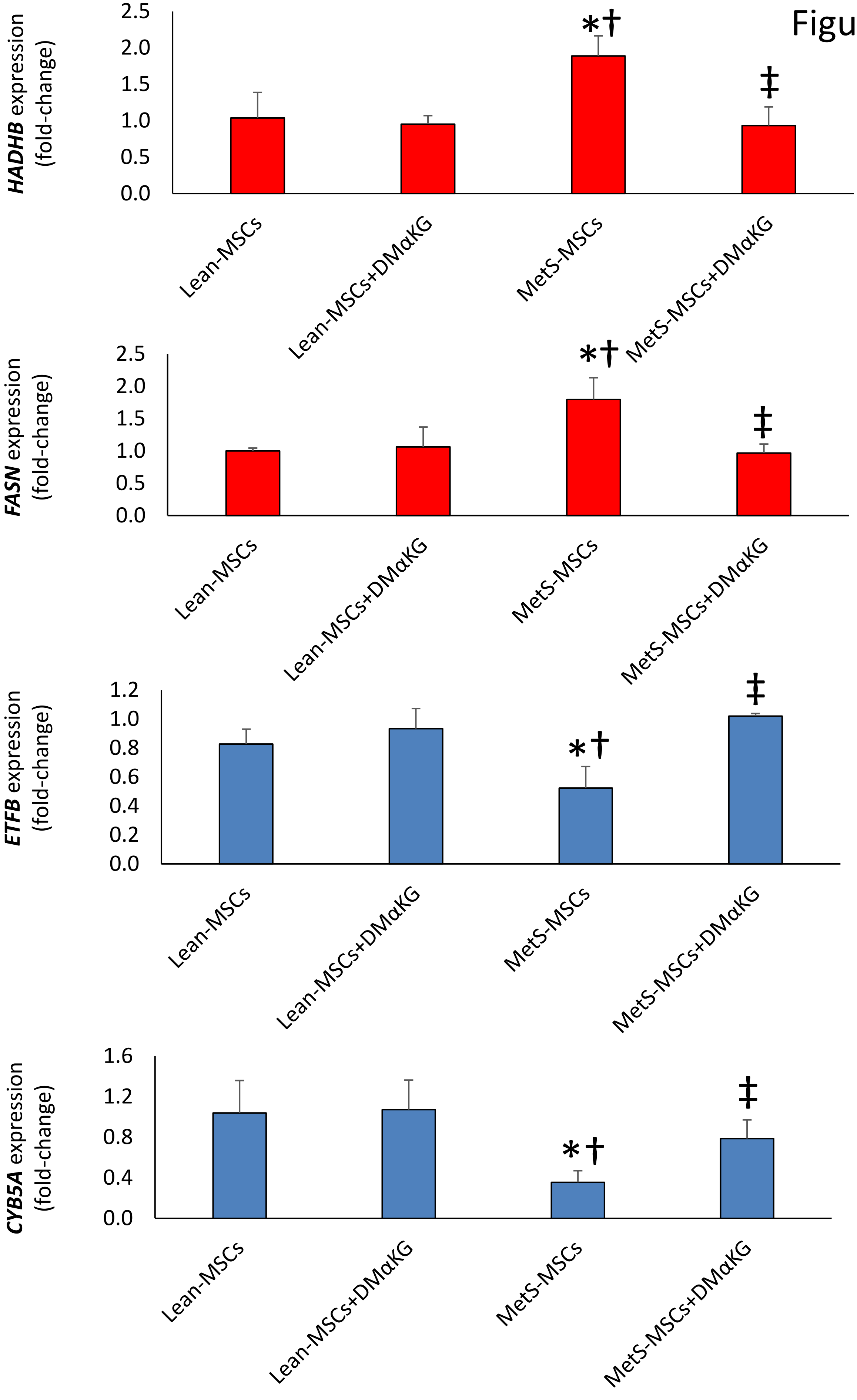

HADHB expression (fold-change)
2.5
2.0
1.5
1.0
0.5
0.0
*†
‡
Lean-MSCs
Lean-MSCs+DMαKG
MetS-MSCs
MetS-MSCs+DMαKG
FASN expression (fold-change)
2.5
2.0
1.5
1.0
0.5
0.0
*†
‡
Lean-MSCs
Lean-MSCs+DMαKG
MetS-MSCs
MetS-MSCs+DMαKG
ETFB expression (fold-change)
1.2
1.0
0.8
0.6
0.4
0.2
0.0
*†
‡
Lean-MSCs
Lean-MSCs+DMαKG
MetS-MSCs
MetS-MSCs+DMαKG
CYB5A expression (fold-change)
1.6
1.2
0.8
0.4
0.0
*†
‡
Lean-MSCs
Lean-MSCs+DMαKG
MetS-MSCs
MetS-MSCs+DMαKG

Figure S11

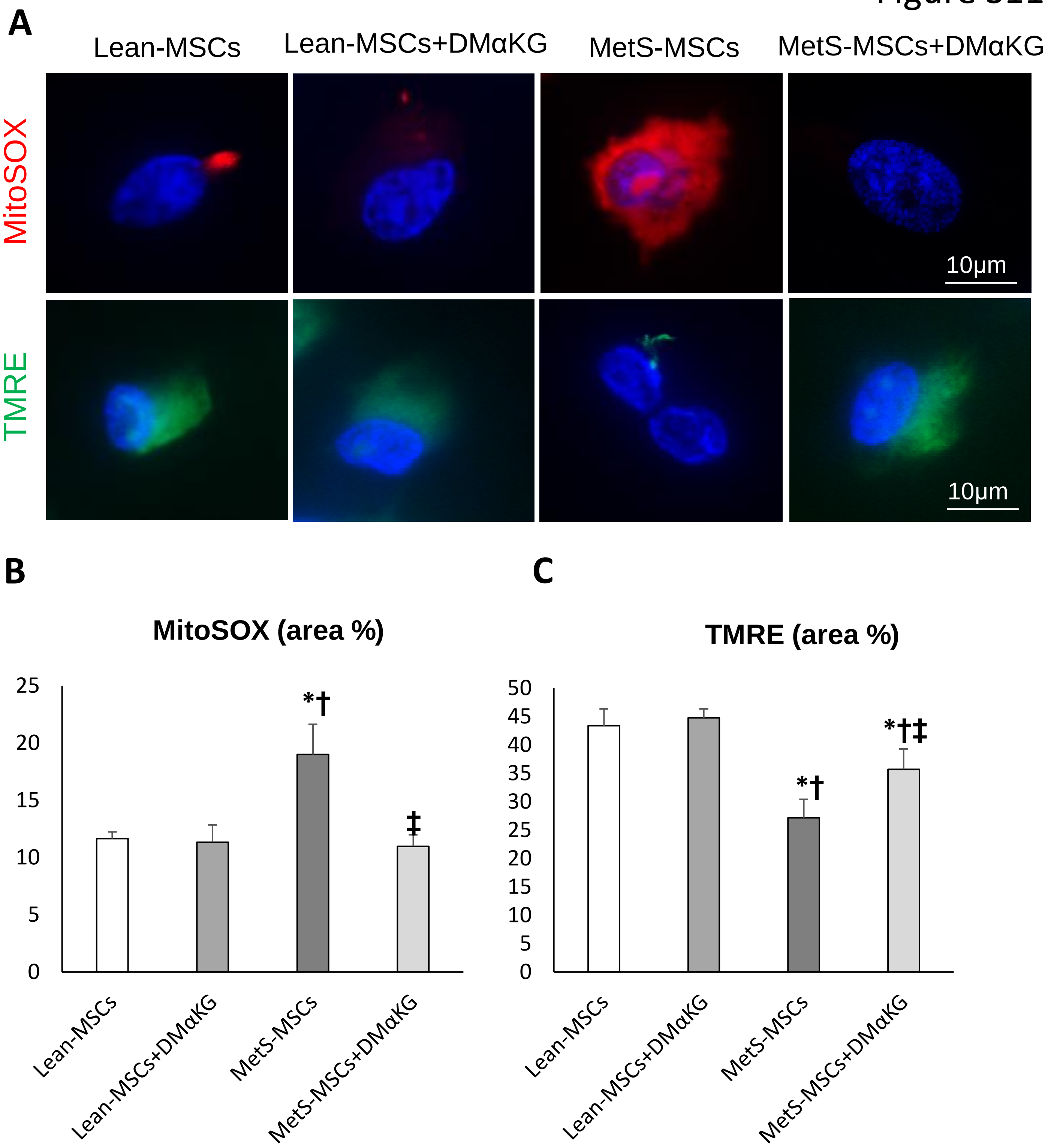
A
Lean-MSCs
Lean-MSCs+DMαKG
MetS-MSCs
MetS-MSCs+DMαKG
MitoSOX
TMRE
10μm
10μm
B
MitoSOX (area %)
25
20
15
10
5
0
*†
‡
Lean-MSCs
Lean-MSCs+DMαKG
MetS-MSCs
MetS-MSCs+DMαKG
C
TMRE (area %)
50
45
40
35
30
25
20
15
10
5
0
*†
*†‡
Lean-MSCs
Lean-MSCs+DMαKG
MetS-MSCs
MetS-MSCs+DMαKG
